# Functional Precision Oncology Approach Using Nanoliter Droplet Array for Drug Sensitivity Testing in Lung Cancer

**DOI:** 10.1002/adhm.202503761

**Published:** 2026-05-15

**Authors:** Maryam Salarian, Vicky L. Schiling, Thomas Muley, Michael Allgäuer, Florian Eichhorn, Michael Meister, Marc A. Schneider, Anna A. Popova

**Affiliations:** ^1^ Institute of Biological and Chemical Systems–Functional Molecular Systems (IBCS–FMS) Karlsruhe Institute of Technology (KIT) Eggenstein–Leopoldshafen Germany; ^2^ Translational Research Unit (STF) Thoraxklinik at Heidelberg University Hospital Heidelberg Germany; ^3^ Translational Lung Research Center Heidelberg (TLRC) Member of the German Center for Lung Research (DZL) Heidelberg Germany; ^4^ Institute of Pathology Heidelberg University Hospital Heidelberg Germany; ^5^ Department of Thoracic Surgery Thoraxklinik at Heidelberg University Hospital Heidelberg Germany

**Keywords:** droplet microarray, drug sensitivity and resistance test (DSRT), functional precision oncology, lung cancer

## Abstract

Functional precision oncology aims to support personalized cancer therapy by assessing drug sensitivity in patient‐derived tumor cells ex vivo. However, conventional drug sensitivity and resistance testing (DSRT) platforms typically require large numbers of cells, limiting their applicability to surgically resected tumors and posing challenges for patients diagnosed at advanced stages, where only small biopsy samples are available. To address this limitation, we developed a miniaturized DSRT workflow based on a Droplet Microarray (DMA) chip, which comprises 672 hydrophilic spots separated by superhydrophobic borders and enables high‐throughput screening in nanoliter volumes. Using 300 cells per 200‐nL droplet, lung cancer cells freshly isolated from surgical specimens were tested against 12 compounds across five concentrations and five replicates (360 experimental conditions). This required approximately 120,000 cells total, including additional cells for handling and processing. The approach generated drug‐specific dose–response profiles and variable IC_50_ values across tumors of the same subtype. Comparable drug responses were also observed across three spatially distinct regions of the same tumor, indicating consistent assay performance. Overall, these results demonstrate that DSRT on the DMA platform is feasible with limited numbers of cells derived from clinical samples and may be useful for functional drug testing when tissue availability is constrained.

## Graphical abstract

A miniaturized drug sensitivity and resistance testing (DSRT) workflow based on the Droplet Microarray (DMA) platform enables functional drug testing using minimal patient‐derived tumor material. By screening nanoliter‐scale droplets containing as few as 300 cells, this approach generates reproducible and tumor‐specific drug response profiles, demonstrating feasibility for precision oncology applications when tissue availability is limited.

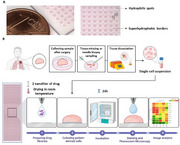

## 1 Introduction

Precision oncology aims to provide optimal therapy at the right time for each patient by considering their health history, genetic profile, and lifestyle, thereby maximizing treatment efficacy and minimizing side effects [1]. One approach in precision oncology is molecular profiling, which identifies cancer‐related alterations that can help predicting therapeutic responses or guide targeted treatment decisions [2]. In the targeted therapy approach, the drugs interfere with molecular targets involved in cancer growth, survival, and proliferation. However, the correlation between specific mutations and effective treatments remains incomplete [3]. Large‐scale studies such as NCI‐MATCH [4], I‐PREDICT [5], and WINTHER have demonstrated the potential and the current limitations of genomics‐driven treatment selection. NCI‐MATCH reported low response rates in many treatment arms, ranging from 0% to 38%, highlighting the limited number of patients with actionable mutations that respond to existing therapies. In contrast, I‐PREDICT showed that matching more molecular alterations to targeted therapies was associated with improved clinical outcomes, with a clinical benefit rate of 56%. WINTHER trial, which integrated transcriptomic and genomic profiling, reported a clinical benefit rate of 35% [6]. The limitations of molecular profiling alone can be due to the absence of druggable mutations, tumor heterogeneity, and development of resistance [3]. To overcome these challenges, functional precision oncology offers a complementary strategy by guiding therapy selection based on the actual drug response of individual tumors tested ex vivo [7]. Drug Sensitivity and Resistance Testing (DSRT) involves treating cancer cells originating from biopsies with a variety of anticancer drugs in vitro with a goal to determine the most effective treatment for each patient. This test can identify the individual sensitivities of tumors to different therapies, aiding in the design of personalized treatments for each patient [8, 9].

Lung cancer is the most common cancer worldwide, with more than 1.8 million deaths in 2020 [10]. Most of the cases are diagnosed at a late stage of the disease, resulting in poor 5‐year overall survival rates of less than 30% despite a variety of newly introduced therapies in the last decade [11]. Moreover, more than 20% of patients with complete surgical resection show a later recurrence of the disease [12]. For cases with lymph node involvement, adjuvant therapy following surgical resection is considered standard of care [13]. However, the efficiency of these adjuvant treatments is poor to evaluate since lung tumors are often heterogeneous and metastases can still be diagnosed several years after initial surgical resection [14].

In functional precision oncology, patient‐derived models are used to study lung cancer, predict treatment responses, and develop personalized therapies. One approach uses short‐term monolayer cultures in microtiter plates, allowing rapid cell expansion and drug screening. For example, Kim et al. showed that drug sensitivity profiles of lung cancer cells cultured in 384‐well plates (1000 cells/well) could predict clinical responses and reveal resistance mechanisms beyond genomic testing [15]. Another strategy involves 3D organoids, which preserve tumor architecture and heterogeneity, although they lack the full tumor microenvironment, such as immune and stromal components [16]. Kim et al. generated organoids from five lung cancer subtypes that reflected the histological and genetic features of the original tumors and showed patient‐specific drug responses [17]. Ding et al. developed microfluidic‐based Micro‐Organospheres (MOS), incorporating ∼30 cells per hydrogel droplet, and screened them with 119 FDA‐approved drugs. Obtained drug responses correlated with clinical outcomes and retained aspects of the tumor immune microenvironment [18].

Despite its potential, DSRT is not yet widely recommended as a first‐line prognostic method for therapy selection in clinical settings. Functional precision oncology requires a sufficient number of viable patient‐derived cells, which can be difficult to obtain from small samples [7]. As a result, in vitro tests primarily rely on tumor cells from surgical resections, restricting sensitivity profiling to operable patients. However, most lung cancer cases are diagnosed at an advanced, inoperable stage, where biopsies are collected for diagnostics [19]. While core needle biopsies (CNB) provide tissue for pathology and genetic profiling, it rarely yields enough viable cells for DSRT. In some cases, CNB‐derived cells can be expanded in vitro (e.g., as organoids) or in vivo (e.g., in PDX models), but these approaches are expensive, not successful for all patients, and time‐consuming, often taking weeks or even months, time that many cancer patients cannot afford [7, 8, 18]. Therefore, these tests have primarily been conducted on cancer types where a large number of patient tumor cells can be obtained, such as hematologic cancers [20, 21]. Additionally, high reagent and compound costs are another challenge, particularly for expensive targeted therapies and large compound libraries used in high‐throughput screening [8].

Miniaturization technologies such as the Droplet Microarray (DMA) offer a promising solution by enabling comprehensive and efficient drug screening using reduced cell numbers and reagent volumes, thus maximizing the utility of small biopsy samples. The miniaturized DMA chip is the size of a standard microscope slide and features an array of 672 hydrophilic spots separated by superhydrophobic borders. It has been used previously to culture different cell types [22, 23], and its potential for DSRT on primary patient‐derived chronic lymphocytic leukemia (CLL) cells has also been investigated [24]. The hydrophilic–superhydrophobic surface pattern enables the formation of hundreds of isolated nanoliter‐scale droplets (∼200 nL) suitable for cell culture and drug screening [25, 26]. Compared to standard drug sensitivity and resistance testing (DSRT) performed in 384‐well plates, which typically require reaction volumes of 30–50 µL, this platform reduces reagent consumption by approximately 150–250‐fold. In parallel, the required cell input per condition is reduced from approximately 1000–5000 cells to about 100–300 cells. This reduction in volume and cell requirements is particularly relevant for biopsy‐derived samples, especially those obtained from core needle biopsies (CNB), where cell numbers are often limited. Under these constraints, the same amount of patient‐derived material can be allocated to approximately 3‐ to 50‐fold more experimental conditions compared to conventional microtiter plate–based assays, depending on the initial cell yield. This increase in assay capacity is important for the potential future clinical application of functional drug testing, as it allows a substantially broader range of drug conditions to be assessed from limited patient‐derived material using the droplet microarray (DMA) platform.

In this study, we demonstrate for the first time the feasibility of culturing and functionally screening freshly derived cells from solid tumors, specifically lung cancer samples obtained from surgical resections and core needle biopsies—using a highly miniaturized droplet‐based format. We show that viable, heterogeneous primary tumor cell populations can be maintained on the droplet microarray (DMA) platform and yield robust, reproducible drug response readouts without prior in vitro expansion. Using a focused panel of clinically relevant chemotherapeutic agents, we observed drug‐specific and patient‐dependent response patterns, demonstrating that quantitative functional screening is compatible with low‐input primary material. Together, this work establishes an end‐to‐end DSRT workflow that prioritizes efficient use of limited, non‐expanded patient‐derived cells and enables substantially broader experimental conditions than conventional formats. While further clinical validation will be required, these results support miniaturized functional assays as a practical strategy for drug sensitivity testing in settings where tissue availability is inherently constrained.

## 2 Materials and Methods

### 2.1 Ethics and Tissue Acquisition

This study was conducted in accordance with the University of Heidelberg Ethics Committee of the Medical Faculty (Approval No: S‐270/2001, title: Heidelberg Lung Biobank: Biospecimens and data for biomedical research to improve diagnostics as a basis for individualized therapy for lung diseases) and the Ethics Committee of KIT. All patients provided written informed consent prior to surgery, allowing the use of their tissue samples for research purposes. The consent process included detailed information about the study's aims and potential risks. Patients were assured that their participation was voluntary, and their identity would remain confidential.

Lung tumor tissues were obtained from patients diagnosed with non‐small cell lung carcinoma (NSCLC) at operable stages I‐III, undergoing surgical resection at Thoraxklinik Heidelberg (University Hospital of Heidelberg, Germany). The patients were not treated with any inductive therapy before surgery. Samples were collected within 24 h post‐surgery, immediately processed for research purposes, and provided by Lung Biobank Heidelberg, a Member of the BioMaterialBank Heidelberg (BMBH) and the biobank platform of the German Center for Lung Research (DZL).

### 2.2 Culturing Cells on DMA Chip

Droplet‐microarray (DMA) slides (1 mm × 1 mm spots) were purchased from Aquarray GmbH (Eggenstein‐Leopoldshafen, Germany). These chips contained 672 square hydrophilic spots arranged in an array format of 14 × 48 (Catalogue number G‐np‐102). Before seeding, the DMA chips were sterilized with 100% ethanol and dried under a clean bench for 20 min following the manufacturer's instructions. Cell concentration was determined using an automated cell counter (Countess II FL Automated Cell Counter, Thermo Fisher Scientific, USA). Based on the measured concentration, cell suspensions were diluted or concentrated to achieve a final density of 1.5 × 10^6^ cells/mL, corresponding to 300 cells per 200‐nL droplet during dispensing. Cells were seeded onto the DMA chips using an automated non‐contact liquid dispenser, I‐DOT One (Dispendix GmbH, Germany), at a volume of 200 nL per spot. The humidity during dispensing was adjusted to 70% using a humidifier connected to the I‐DOT One. After dispensing, the DMA chip containing cells was immediately placed inside a humidity‐controlled Petri dish. This dish, a 10 cm Petri dish filled with 2 mL of PBS and covered with a lid containing a humidifying pad, was then placed in a standard cell culture incubator (37°C and 5% CO_2_).

### 2.3 Tissue Processing

Tissues were kept at 4°C in MACS Tissue storage solution (Miltenyi Biotec, Bergisch Gladbach, Germany) until dissociation, which was performed within 24 h after surgery. Manual single‐cell suspensions were performed as follows: tissues were transferred to a 10 cm Petri dish and cut into ∼ 1 mm^3^ pieces using sterile scalpels. The tissue pieces were then transferred to a 50 mL Falcon tube and pipetted repeatedly until a homogeneous suspension was achieved. The suspension was centrifuged for 10 min at 300 × g at room temperature. Liberase DH (0.28 Units/mL) (Roche, Switzerland) in DMEM medium was added to the pellet and incubated for 2 h on an overhead rotator at 37°C. Cells were filtered using 100 µm and subsequently 40 µm cell strainers (Corning, USA) and centrifuged at 300 × g for 10 min. The cells were then resuspended in PBS and loaded on top of Histopaque (Sigma–Aldrich, USA), followed by centrifugation for 20 min at 1800 × g without brake at room temperature. The separated cells were diluted with PBS and centrifuged again for 5 min at 300 × g.

For semi‐automated tumor dissociation, the human Tumor Dissociation Kit (Miltenyi Biotec, Germany) was used. The procedure followed the manufacturer's protocol using the gentleMACS Octo Dissociator with Heaters (Miltenyi Biotec, Germany). Tumor samples were minced with two scalpels into 2–4 mm fragments and placed into gentleMACS C Tubes (Miltenyi Biotec, Germany) containing the enzyme mix and DMEM medium. These tubes were then placed onto the gentleMACS Octo Dissociator with Heaters, and the “37C_h_TDK_2” program was run. The cell suspension was filtered through 100 and 40 µm cell strainers, loaded on top of Histopaque, and diluted with PBS as previously described. Cells were maintained in DMEM/F‐12 (Gibco, USA) without glutamine, supplemented with GlutaMAX (Gibco, USA), 1% penicillin/streptomycin (Gibco, USA), 3 µg/mL of ROCK inhibitor (Y‐27632, Stemcell Technologies, Canada), and Airway Epithelial Cell Growth Medium Supplement Pack (C‐39160, PromoCell, Germany): 0.004 mL/mL bovine pituitary extract, 10 ng/mL epidermal growth factor, 5 µg/mL insulin, 0.5 µg/mL hydrocortisone, 6.7 ng/mL triiodo‐L‐thyronine, and 10 µg/mL transferrin.

For each tumor specimen, the length, width, and height were measured. The approximate tumor volume was estimated by multiplying these three dimensions (Volume = length × width × height). The number of viable cells isolated from each tumor was then normalized to this volume, and the resulting cell yield per cm^3^ was added to Table S1 to allow comparison of tissue processing efficiency across different samples.

### 2.4 Drug Treatment

The DMA chips were sterilized with 100% ethanol and dried under a clean bench for 20 min. Anticancer compounds in five different concentrations, with five repeats per concentration, were dispensed onto the DMA slides using a sciFLEXARRAYER S11 liquid dispenser (Scienion, Germany). For each drug, 10 mm stock solutions were prepared in DMSO (Sigma–Aldrich, USA). The following concentrations were prepared: 100, 50, 25, 12.5, and 6.25 µm for pemetrexed, cisplatin, carboplatin, and cyclophosphamide; 30, 15, 7.5, 3.75, and 1.87 µm for etoposide; 10, 5, 2.5, 1.25, and 0.62 µm for topotecan; 5, 2.5, 1.25, 0.62, and 0.31 µm for vincristine; 0.5, 0.25, 0.125, 0.06, and 0.03 µm for docetaxel; and 1, 0.5, 0.25, 0.125, and 0.06 µm for vinorelbine, paclitaxel, gemcitabine, and doxorubicin. DMSO (2 nL) was used as a control. The DMA chips containing the pre‐dispensed drugs were dried in the dark at room temperature for 24 h to ensure the evaporation of the DMSO content from the spots. The pre‐dispensed slides were then stored in a dark box containing silica gel (Sigma–Aldrich, Germany) before use. Cells were incubated with the drugs for 24 h before performing staining and viability tests.

### 2.5 Dose–Response Comparison on DMA and 384‐Well Plates

Comparative dose–response analyses were performed using the human lung adenocarcinoma cell line A549 (A549 lung cancer cells were obtained from the American Type Culture Collection ATCC, Manassas, VA, USA). For the DMA platform, 300 cells were dispensed in 200 nL droplets onto pre‐printed compound spots. For conventional assays, 5000 cells per well were seeded in 30 µL medium in 384‐well plates. Cells were incubated with compounds for 48 h under standard culture conditions.

For the DMA readout, cell viability was assessed by live–dead staining followed by automated fluorescence microscopy, as described below. For the 384‐well plates, cell viability was measured using the CellTiter‐Glo luminescence assay (Promega Corporation, Madison, WI, USA) according to the manufacturer's instructions.

Twelve chemotherapeutic agents were tested using 10‐point, twofold serial dilutions. Final concentration ranges (µm) were as follows: pemetrexed, paclitaxel, cisplatin, carboplatin, doxorubicin, cyclophosphamide, vincristine, and vinblastine (100–0.195 µm); gemcitabine and etoposide (40–0.078 µm); docetaxel (16–0.031 µm); and topotecan (32–0.0625 µm). Compounds were tested at identical concentration ranges on both platforms.

### 2.6 Live and Dead Staining and Fluorescent Microscopy

For live/dead staining, 50 nL of a staining solution containing 1 µg/mL Propidium Iodide (PI), 10 µg/mL Hoechst 33342, and 1 µg/mL Calcein‐AM (all from Invitrogen, USA) in PBS (Gibco, USA) was dispensed into each droplet using the I‐DOT dispenser (Dispendix, Germany). The chip was then incubated at 37°C in a standard cell culture incubator for 30 min.

For microscopy, the slide was placed in a 4‐well plate with humidity pads and sealed with parafilm to prevent evaporation during imaging. Cells were examined and imaged using an automated Leica 3D Thunder Imager (Leica, Germany) for both brightfield and fluorescence microscopy.

### 2.7 Evaluation of Tumor Composition

Hematoxylin and Eosin (H&E) stainings were performed during the diagnostic routine by the Institute of Pathology at Heidelberg University Hospital. Tumor specimens were scanned on a slide scanner (Aperio AT2, Leica, Wetzlar, Germany) with 40x magnification, and tumor cell content was evaluated by an experienced lung pathologist based on H&E staining. Tumor cell content was annotated in Figure S2A.

### 2.8 Immunostaining and Flow Cytometry

Cells were washed with PBS (Gibco, USA) to remove the culture medium. The cells were then fixed with 4% paraformaldehyde (PFA, Thermo Fisher Scientific, USA) for 10 min at room temperature. All samples were blocked with a Blocker BSA solution (Thermo Fisher Scientific, USA) for 1 h at room temperature. The samples were then incubated with Alexa Fluor 488‐conjugated anti‐EpCAM antibody (clone EBA‐1, Santa Cruz Biotechnology, USA) at a concentration of 1:200 in 1% BSA overnight at 4°C. The following day, the samples were rinsed with PBS three times. For flow cytometry analysis, stained cells were resuspended in PBS and analyzed using a Guava easyCyte System (Luminex Corporation, USA) with GuavaSoft 4.5 software. Fluorescence compensation was applied to account for spectral overlap, and data analysis, including gating strategy, was performed using FlowJo software (FlowJo, LLC, USA). Quadrant gating was used to distinguish EpCAM‐positive and EpCAM‐negative populations.

### 2.9 Data Analyses

Image analysis for fluorescent images to estimate the number of cells per spot was performed using the ImageJ/Fiji software. The images were first converted to 8‐bit format, followed by manual adjustment of the threshold. To separate individual cells, the “Watershed” algorithm was applied. The “Analyze Particles” function was then used to quantify the number of stained cells in each spot. For automated processing of multiple images, the “Batch Macro” function was employed.

Dose‐response curves were generated using GraphPad Prism (GraphPad Software, LLC, California, USA). Data were entered into an XY data table with five replicate response values for each drug concentration. The drug concentrations (X values) were log‐transformed prior to curve fitting using the “Transform” analysis feature in Prism. Nonlinear regression was performed using the “log(agonist) versus response—Variable slope (four parameters)” model under the “Dose‐response—Stimulation” equation group.

IC50 values were determined by drawing a horizontal line at 50% viability on the dose‐response curve. The intersection of this line with the fitted curve was used to identify the corresponding drug concentration on the log‐transformed X‐axis. This value was then antilog‐transformed to calculate the IC50 in terms of the original drug concentration. Error bars represent the standard deviation derived from the five replicates at each drug concentration.

Statistical analyses were also performed using GraphPad Prism. Error bars indicate standard deviation derived from the five replicates at each drug concentration. For comparisons between two independent groups (e.g., manual vs. semi‐automated tumor dissociation methods), an unpaired two‐sided *t*‐test was used. A *p*‐value less than 0.05 was considered statistically significant. Statistical significance for comparisons of drug responses across different numbers of cells per spot was determined using ordinary one‐way ANOVA, with highly significant differences (^****^ *p* < 0.0001, ^***^ *p* < 0.001, ^**^ *p* < 0.01) for comparisons between different groups, while “NS” indicates a nonsignificant statistical difference between the data groups.

Dose–response curves for the DMA and 384‐well plate experiments were analyzed using the RStudio environment (R version 4.5.2). Data were fitted using nonlinear regression to generate dose–response curves, from which half‐maximal inhibitory concentration (IC_50_) values were calculated. Drug Sensitivity Scores (DSS) were computed to quantify overall drug response by integrating multiple parameters of the dose–response curves, including potency, slope, and maximal effect. DSS values were calculated according to the method described by Yadav et al. [27]. The resulting IC_50_ and DSS values were used to compare drug responses between the DMA and 384‐well plate platforms.

### 2.10 Digital Tools for Figure Creation and Text Editing

Figures were created using BioRender (BioRender.com, Toronto, Canada), an online platform for scientific illustration, to ensure clarity and consistency in visual presentation. ChatGPT (OpenAI, San Francisco, USA) was used as a writing assistant to improve the clarity, structure, and readability of the manuscript text. All content was critically reviewed and revised by the authors to ensure accuracy, scientific rigor, and alignment with the study's objectives.

## 3 Results

### 3.1 Processing of Resected Lung Cancer Tumors for Functional Screening

**FIGURE 1:**
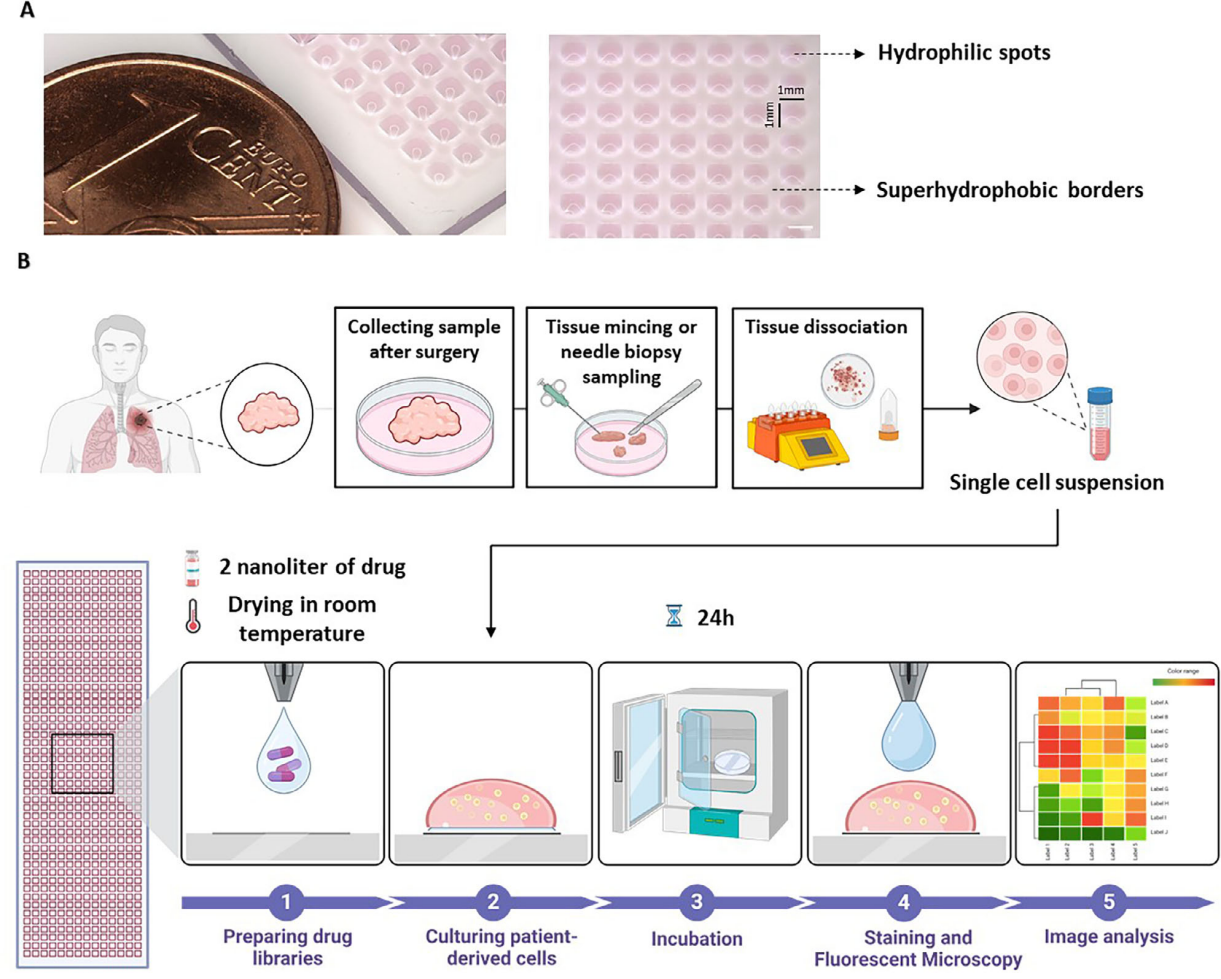
Concept and experimental workflow of miniaturized DSRT for patient‐derived lung cancer cells on the DMA chip. (A) Photo of a part of DMA. Comparison of the size of DMA spots to a 1‐cent coin (left panel). The DMA platform features hydrophilic square‐shaped spots, each measuring 1 mm × 1 mm, separated by superhydrophobic borders containing droplets of cell culture medium (right panel). Scale bar: 1 mm. (B) Scheme of the DSRT workflow. Surgical specimens or artificial needle biopsies derived from surgical material, obtained from patients diagnosed with non‐small cell lung cancer (NSCLC) are received after surgery and dissociated into a single‐cell suspension. (B) Drug libraries are prepared by dispensing 2 nL of drugs in the desired concentration onto each spot of the DMA chip. The slides are dried at room temperature and stored in dark dry conditions. The obtained cell suspension is then dispensed onto the pre‐printed drug libraries, and cells are exposed to the drugs for 24 h. Afterward, the cells are stained with fluorescent dyes Hoechst, Calcein‐AM, and PI, and then imaged using an automated screening fluorescence microscope. The images are analyzed to determine cell viability.

The primary goal of this study was to establish a miniaturized workflow for drug sensitivity testing of freshly isolated primary cells derived from solid tumors, using minimal cell input suitable for application to small clinical specimens such as CNBs. Lung cancer was selected as a model due to its clinical relevance and the frequent reliance on limited biopsy material for diagnosis and treatment decisions. Figure 1 illustrates the concept and workflow of the miniaturized DSRT on DMA. The workflow was optimized on a DMA chip containing an array of 672 square hydrophilic spots, arranged in a 14 × 48 layout, each spot measuring 1 mm × 1 mm (Figure 1A,B). Lung cancer tissue samples were collected, minced, and enzymatically dissociated to a single‐cell suspension for further downstream analyses (Figure 1B). The collected tumors ranged in size from 1.9 cm × 1.8 cm × 0.3 cm (0.96 g) to 3.1 cm × 2.9 cm × 0.4 cm (4.19 g), based on measurements of their largest dimensions (TableS1).

In this study, two methods for tumor tissue dissociation were optimized and compared as part of workflow development. The manual dissociation method was initially used during the early phase of the study and served as a baseline approach before implementation of a semi‐automated system. The first method involved cutting tumor samples, followed by enzymatically digesting the pieces by Liberase DH (0.28 Units/mL) in DMEM medium in a Falcon tube while rotating the sample. Cell count and viability data obtained with this method are shown in Figure S2A and TableS1 for patients 6–11. On average, this method yielded 1.96 × 10^6^ (calculated from six samples, standard deviation: 1.04) with an average viability of 72.66% (standard deviation: 11.5) (Figure S2B,C). The second method is a semi‐automated approach, and involves using the gentleMACS Octo Dissociator with Heaters in combination with the Tumor Dissociation Kit. On average, this method yielded 3.31 × 10^6^ cells (calculated from thirteen samples: Patients 1–5, 12, and 13; standard deviation: 3.76), with an average viability of 75% (standard deviation: 13.72, Figure S2B,C).

To account for variations in tumor size, the number of isolated cells was normalized to tumor volume. The normalized yields confirmed that the semi‐automated dissociation approach increased the yield of isolated cells from tumors by raising the average normalized number of isolated cells from 1.96 × 10^6^ to 4.75× 10^6^ cells (Figure S2B). This improvement is likely due to the gentleMACS Dissociator's ability to provide consistent and controlled mechanical dissociation across samples, which, when combined with enzymatic digestion, ensures thorough and efficient tissue dissociation.

These results indicate that cell viability was consistently high across surgical materials after tumor dissociation, ranging from 53% to 98%, with an average of 74.26% across tumor samples. Furthermore, between 0.71 × 10^6^ and 13.8 × 10^6^ cells were successfully isolated from each tumor, providing sufficient quantities for drug sensitivity testing (Figure S2). The observed variation in the number of cells isolated from each tumor likely reflects differences in tumor size, cellular density, and tissue characteristics, such as the presence of necrotic cells.

As a next step, we have characterized the obtained cell suspension for the presence of cancer cells after isolation and in vitro culture. Identifying cancer cell biomarkers for all three subtypes of NSCLC is challenging. Lung tumors are highly heterogeneous, with different regions or cells within the same tumor exhibiting varied expression of biomarkers. This intra‐tumor heterogeneity makes it challenging to rely on a single biomarker to identify cancer cells accurately across samples [28]. Additionally, not all biomarkers are compatible with all detection techniques, such as flow cytometry. EpCAM (epithelial cell adhesion molecule) is a well‐known tumor‐associated antigen expressed on healthy epithelial cells, tumors of epithelial origin, circulating tumor cells, and cancer stem cells [29]. To confirm the presence of epithelial cells, representing the tissue of origin of the cancer, and to assess their integrity during tissue dissociation and persistence after in vitro culture, the isolated cells were stained with an anti‐EpCAM antibody, a marker of epithelial lineage, and analyzed by flow cytometry. One fraction of cells was stained immediately after isolation, and another fraction was cultured for 24 h and then stained to ensure EpCAM‐positive cells were not lost during the incubation period. However, it is important to keep in mind that EpCAM expression can be low in lung cancer tumors [30], and healthy epithelial cells also express EpCAM.

The results (Figures S3 and S4) indicate that the proportion of EpCAM‐positive cells ranged from 71.6% to 83.9% immediately after cell isolation, and from 67.4% to 93.4% after 24 h of incubation, confirming that epithelial cells were present in the cell suspension, even after 24 h of incubation. The observed increase in EpCAM‐positive cells after 24 h may be due to the selective survival of epithelial cells in culture or loss of more fragile stromal and immune cells during incubation and washing steps. The estimated tumor cell content in the original tumor pieces prior to dissociation is listed in Figure S2A, with an average of approximately 40% across all samples used in this study. The high percentage of EpCAM‐positive cells observed in the dissociated suspensions compared to the estimated tumor content in the original tumor tissue likely reflects preferential dissociation or survival of epithelial cells, including both tumor and non‐tumor populations. The fact that we used not exactly the same piece of the tumor for our analyses as for routine diagnostic might also influence the distribution of the cell populations to some degree.

### 3.2 Drug Sensitivity Testing on Patient‐Derived Lung Cancer Cells

As a next step, we proceeded to drug sensitivity testing after confirming that patient‐derived lung cancer cells remained viable after dispensing on the DMA chip. The concentration of isolated cells was adjusted to achieve 300 cells per 200‐nL droplet. To evaluate cell viability and variability of controls, cells isolated from Patient 11 were cultured on 134 spots of the DMA chip. Patient 11 was chosen based on sample availability during assay optimization and was not selected due to any specific biological or clinical features. After 24 h of incubation, cell viability was assessed by dividing the number of live cells (Calcein‐positive) by the total number of cells (Calcein‐positive + PI‐positive), as shown in Figure S5. The results confirmed that cells remained viable after dispensing on the DMA spots, validating the suitability of the miniaturized platform for subsequent drug sensitivity testing.

To perform drug sensitivity tests on patient‐derived cells, drug libraries were prepared. Twelve anticancer chemotherapeutic agents commonly used in lung cancer treatment over the past 5 years were selected and dispensed onto a DMA chip, followed by compound drying and subsequent seeding of cells onto the pre‐printed libraries. To confirm that the drugs retained their activity after drying on the DMA slide, we performed a comparative dose–response analysis using both the DMA platform and conventional 384‐well plates with a state‐of‐the‐art protocol using the A549 lung cancer cell line. As shown in Figure S11, the IC_50_ values and DSS (Drug Sensitivity Score) obtained from both platforms were in good agreement for all 12 drugs. The approach of pre‐printed libraries on DMA enables the rapid generation of disease‐specific drug panels that can be readily applied when patient‐derived cells become available. The feasibility of this method was first demonstrated in Popova et al. [24], and has since been applied consistently across multiple high‐throughput screening studies [31, 32]. Five different concentrations of drugs, in five replicates for each concentration (Table 1), were selected based on their IC50 distribution for lung adenocarcinoma and lung squamous cell carcinoma, obtained from the Genomics of Drug Sensitivity in Cancer (GDSC) database [33]. Details of the drug names, mechanisms of action, and concentrations used in this study are provided in Table 1. Following library preparation on DMA, cells were dispensed onto pre‐printed DMA libraries and cultured for 24 h. In line with commonly used approaches for testing primary cell suspensions with the CellTiter‐Glo (CTG) assay [34], we instead assessed cell viability by live/dead staining of the entire cell population. Viability was then compared across cells exposed to different drug concentrations to generate dose–response curves.

**TABLE 1:** Anticancer cytotoxic drugs used in this study, including their mechanisms of action and the concentrations (in µm) at which they were tested. The concentrations range from C1 (lowest concentration) to C5 (highest concentration). These concentrations were created through a 2‐fold serial dilution, with each subsequent concentration being half of the previous one, maintaining a consistent dilution factor.

Drug	Mechanism of action	Concentration (µM) C1/C2/C3/C4/C5	Reference
Vinorelbine	Inhibition of microtubule polymerization	0.06/0.125/0.25/0.5/1	[35]
Pemetrexed	Inhibition of thymidylate synthase	6.25/12.5/25/50/100	[36]
Paclitaxel	Stabilization of microtubules, preventing depolymerization	0.06/0.125/0.25/0.5/1	[37]
Gemcitabine	Inhibition of DNA synthesis	0.06/0.125/0.25/0.5/1	[38]
Docetaxel	Stabilization of microtubules, preventing depolymerization	0.03/0.06/0.125/0.25/0.5	[39]
Etoposide	Inhibition of DNA topoisomerase II	1.8/3.6/7.2/14.4/28.8	[40]
Cisplatin	DNA cross‐linking	6.25/12.5/25/50/100	[41]
Carboplatin	DNA cross‐linking	6.25/12.5/25/50/100	[42]
Vincristine	Inhibition of microtubule polymerization	0.3/0.6/1.25/2.5/5	[43]
Topotecan	Inhibition of DNA topoisomerase I	0.6/1.25/2.5/5/10	[44]
Doxorubicin	Inhibition of DNA topoisomerase II, intercalation into DNA	0.06/0.125/0.25/0.5/1	[45]
Cyclophosphamide	Alkylation	6.25/12.5/25/50/100	[46]

#### 3.2.1 Reproducibility of Drug Sensitivity Testing Across Different Numbers of Cells Per Spot

**FIGURE 2:**
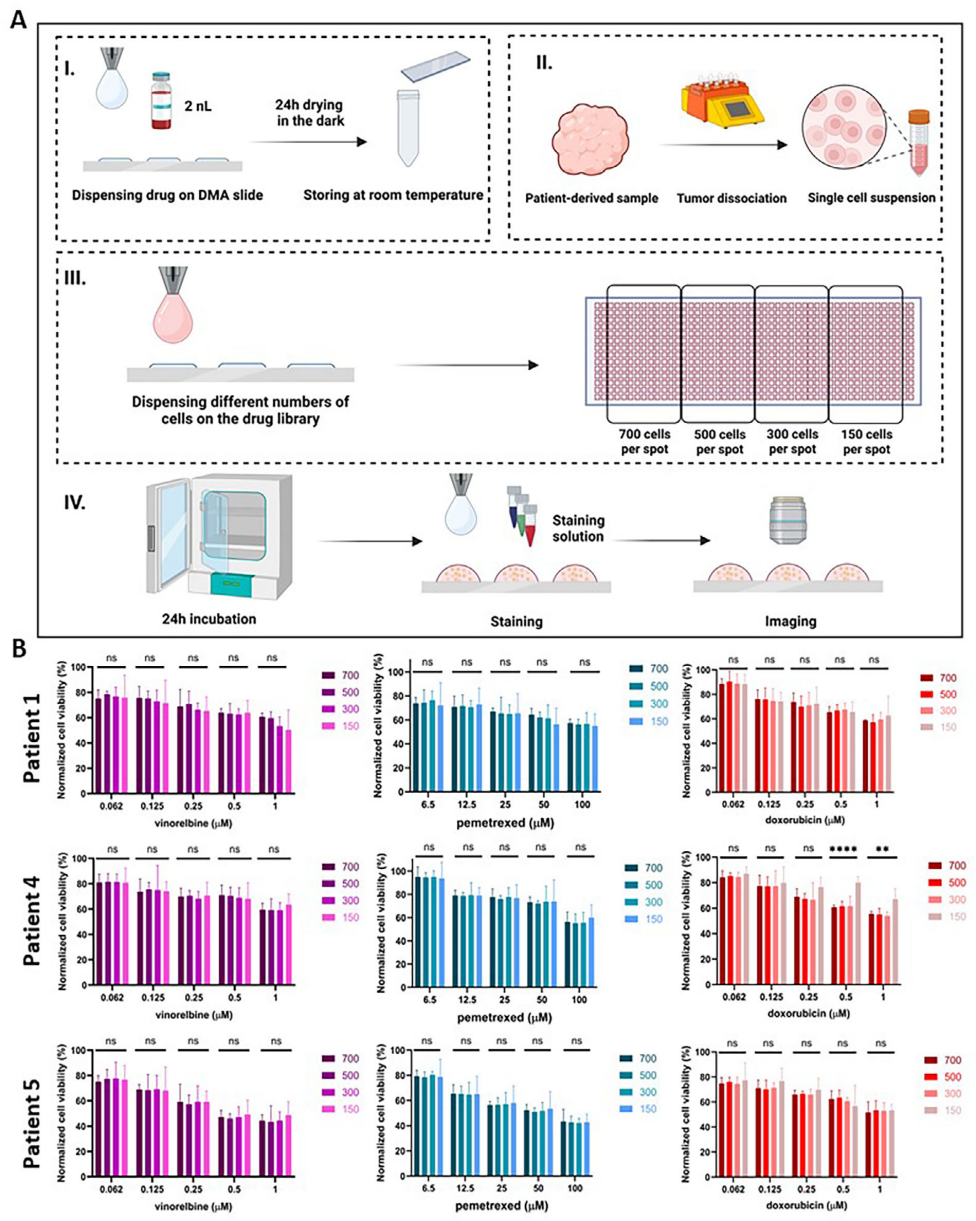
Reproducibility of drug sensitivity testing across different numbers of cells per spot. (A) Workflow for evaluating the reproducibility of drug sensitivity tests on patient‐derived cells. Preparation of drug libraries by dispensing four arrays (11 × 14) of vinorelbine, pemetrexed, and doxorubicin at five concentrations with five replicates for each concentration on a single slide (I). Dissociation of tumor tissues (II) to generate a cell suspension using the semi‐automated method. Dispensing of indicated cell numbers onto an array of 11 × 14 on the DMA chip containing the drug libraries (III). Incubation of cells with drugs for 24 h, followed by staining and imaging to assess cell viability and drug response reproducibility (IV). (B) Overview of cell viability of three exemplary patient‐derived cell suspensions after treatment with the indicated drugs and cell numbers. The cell viability of cells was normalized to the DMSO control. Each drug was tested by five biological replicates. Error bars indicate standard deviation. Statistical significance was determined using one‐way ordinary ANOVA, with highly significant differences (^****^ *p* < 0.0001, ^***^ *p* < 0.001, ^**^ *p* < 0.01) for comparisons between different groups, while “ns” indicates non‐significant statistical differences.

To evaluate how the number of cells per spot can influence reproducibility, variability, and statistics of drug responses in drug sensitivity and resistance tests on the DMA chip, a dedicated workflow was designed, as illustrated in Figure 2A. The tested range of 150–700 cells per spot was selected to reflect low‐to‐moderate cell numbers realistically obtainable from patient‐derived lung cancer samples, including small surgical specimens and needle biopsies, and to identify the lower boundary at which assay variability increases. Drug libraries were prepared on the DMA chip (Figure 2A, Step I), and tumors were dissociated to receive isolated cells, which were added using specific cell counts (Figure 2A, Step II). Cell numbers were adapted to different concentrations and dispensed into DMA containing the drugs at varying concentrations. (Figure 2A, Step III). After incubating the cells with the drugs for 24 h, the cells were stained and imaged as described previously (Figure 2A, Step IV).

Figure 2B shows the results of drug sensitivity tests conducted using different numbers of cells per spot from three patient‐derived samples. Based on our experience, we typically use 150–500 cells per spot on the DMA platform, depending on the cell line. In the current study, we evaluated 700, 500, 300, and 150 cells per spot. Across all conditions, cell viability decreased consistently with increasing drug concentrations. The similarity of the graphs for 700, 500, and 300 cells per spot suggests that the drug testing platform delivers consistent and reproducible results at these levels. The effect of the number of cells per spot on cell viability was generally not significant (*p* = 0.2721–0.9994), except for samples from Patient 4 exposed to doxorubicin (0.5 µm, *p* < 0.0001 and 1 µm, p = 0.0030). This may be attributed to higher variability observed at 150 cells per spot, indicating that assay performance may be more sensitive to cell number below 300 cells per spot. Since no significant differences in drug response reproducibility were observed between 700, 500, and 300 cells per spot, 300 cells per spot was selected as the minimal working cell number for all subsequent experiments. This choice balances statistical robustness and reproducibility with minimal cell consumption, making it suitable for functional drug testing of low‐input clinical samples while maintaining response profiles comparable to higher cell numbers.

#### 3.2.2 Miniaturized Drug Sensitivity Testing and Patient‐Specific Responses

**FIGURE 3:**
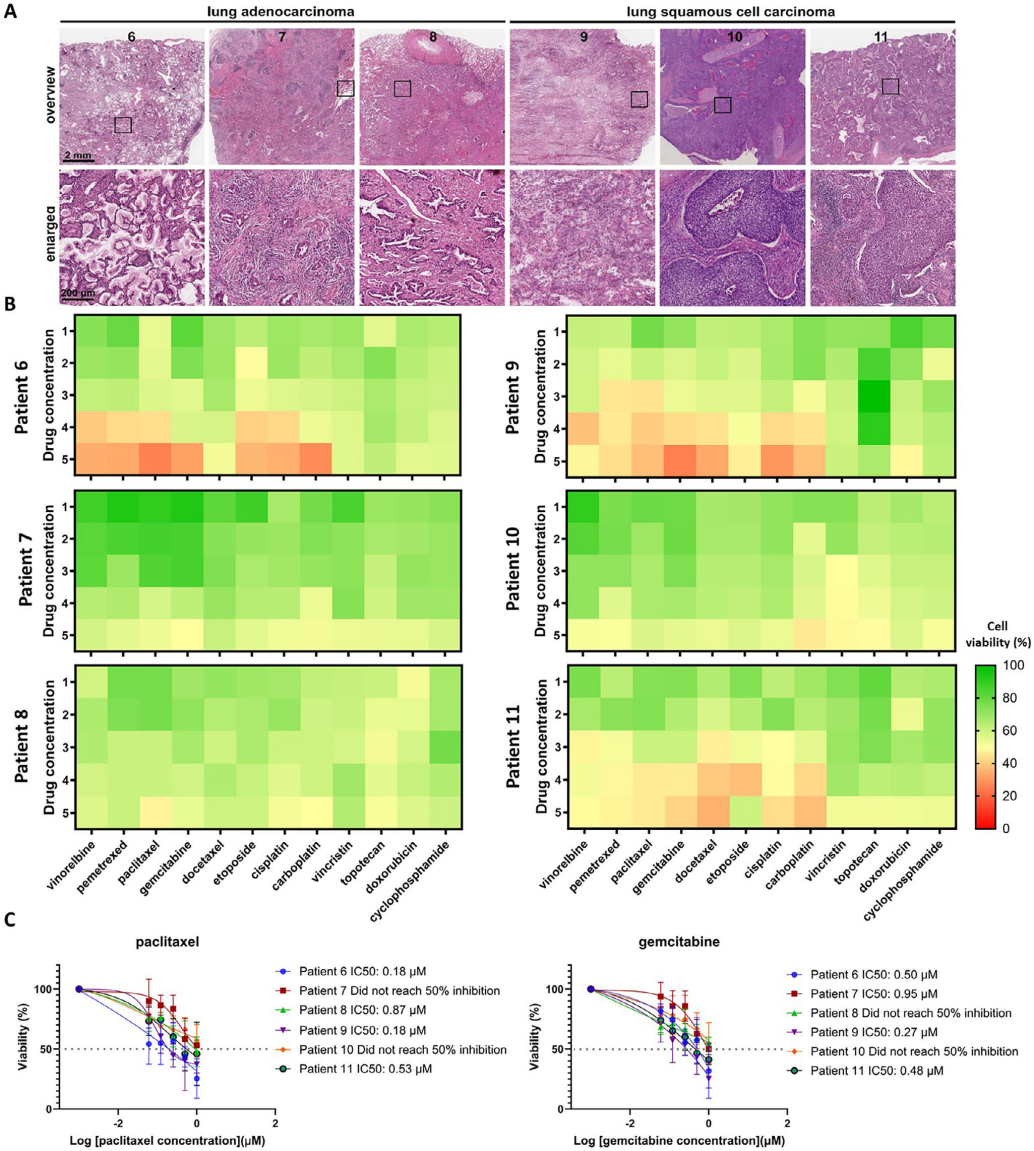
Drug sensitivity test on patient‐derived cells. (A) Representative H&E staining of the tumor samples from Patients 6–11. Scale bars: 2 mm (upper panel) and 200 µm (lower panel). (B) Heat‐maps showing drug sensitivity results for the six samples after 24 h of drug incubation. Cells were stained with a solution containing Hoechst 33342, Calcein‐AM, and PI. Drug concentration 1 represents the lowest concentration, and Drug concentration 5 represents the highest (see also Table 1). (C) Dose‐response curves of paclitaxel (left panel) and gemcitabine (right panel). The IC50 (half‐maximal inhibitory concentration) of each drug was determined. The average was taken from five repeats, with error bars indicating standard deviations.

Chemotherapy targets not only cancer cells but also affects the broader cellular environment of the tumor, which plays a critical role in shaping therapeutic response. The interactions between cancer cells and other cellular components, such as immune cells, fibroblasts, and endothelial cells, are known to influence drug sensitivity and resistance [47, 48]. Therefore, in this study, we used the unsorted cell suspension obtained after tumor dissociation for drug testing, rather than isolated or expanded tumor cell populations. While this suspension was enriched for epithelial cells (70%–80% according to our results), it retained other cell types from the tumor (20%–30%). We were interested in testing the entire cell population rather than pre‐sorted epithelial cells for two main reasons: first, non‐epithelial cells could influence drug responses in vitro; and second, the presence of immune cells allows for the possibility of testing immunotherapy approaches in the future. A systematic comparison of drug responses in pure versus mixed cell populations was beyond the scope of this feasibility study and represents an important direction for future work. In total, 2 × 10^5^ cells were used per library to test responses to 12 drugs at five concentrations. The isolated cells were dispensed onto the drug library, incubated, stained, and imaged to assess viability. In Figure 3B, the total cell viability is shown for cells treated with increasing drug concentrations. The left panel shows drug response data for patients with adenocarcinoma, while the right panel displays data for patients diagnosed with squamous cell carcinoma. Cells from Patients 6, 9, and 11 exhibited higher drug‐induced cell death compared to cells from Patients 7, 8, and 10 across the tested drug concentrations. In contrast, cells from Patients 7, 8, and 10 showed comparatively greater resistance to drug treatment. To study these observations more closely, dose‐response curves for each drug were generated using data from cell viability assays after 24 h of drug incubation (Figure 3C and Figure S6). In line with results from Figure 3B, the IC50 for vinorelbine in Patient 6 was 0.4 µM, whereas for Patients 7 and 8, 50% inhibition was not achieved within the tested concentration range. Additionally, Patients 9 and 11, both diagnosed with stage IIB squamous cell carcinoma, displayed similar responses to some drugs, such as carboplatin and cisplatin, with IC50 values of 25.1 and 24.5 µm, respectively. However, they showed variation in response to doxorubicin. The IC50 of doxorubicin was 1 µm in cells from Patient 9, whereas 50% inhibition was not reached in cells from Patient 11 within the tested concentration range.

Taken together, our data demonstrated the feasibility of testing patient‐derived specimens and suggest an individualized patient response to our tested drugs.

#### 3.2.3 Reproducibility of Drug Sensitivity Testing Within a Single Tumor

**FIGURE 4:**
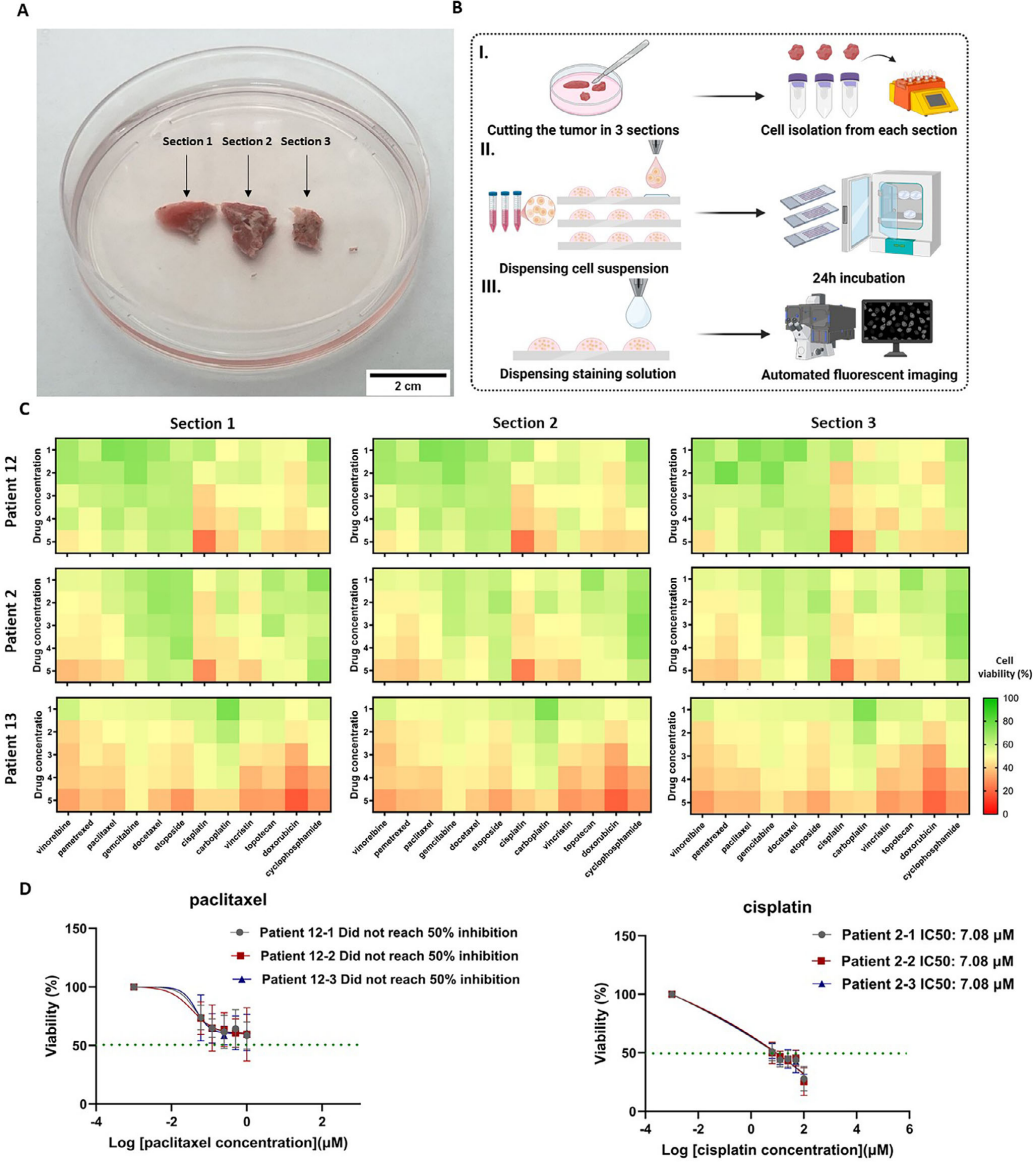
Drug sensitivity across different regions of a single tumor specimen. (A) Each tumor was divided into three sections. (B) Schematic workflow of the drug sensitivity test on three regions of a single tumor. (C) Heatmap of cell viability after 24 h of incubation with the indicated drugs. Each heatmap represents the drug responses of one tumor section: left (1), center (2), and right (3). D) Dose‐response curves generated using data from cell viability assays after 24 h of drug incubation. Viability percentages at different drug concentrations were plotted, and the curves were fitted using nonlinear regression analysis. The IC50 for each section was calculated from five repeats. Error bars represent standard deviations.

To assess the reproducibility of the established methodology, we generated three sections from a single tumor specimen (Figure 4A) and collected isolated cells in separate tubes (Figure 4B, Step I). Each cell population was then tested against a library of 12 anticancer drugs at five different concentrations using the DMA platform (Figure 4B, Step II). Drug responses were evaluated after 24 h of incubation by staining and imaging (Figure 4B, Step III).

The results demonstrated consistent drug sensitivity profiles across the three tumor sections, indicating that intra‐tumoral sampling does not introduce significant variability in assay outcomes (Figure 4C). The dose‐response curves for each drug were generated using data from cell viability assays after 24 h of drug incubation, comparing the responses of three different sections of a single sample to the same panel of drugs (Figure 4C and Figures S7–S9). The dose‐response curves for all tested drugs on three tumor sections are provided in Figures S7–S9. The IC50 was determined from the dose‐response curves. For example, cells isolated from Patient 12 exposed to paclitaxel did not reach IC50 in any of the three sections, whereas cells from Patient 2 exposed to cisplatin consistently reached an IC50 value of 7.08 µm across all three sections. These results demonstrate the reproducibility of drug responses across different regions of the same tumor, which confirms the robustness of the established methodology of cell isolation and miniaturized drug testing on the DMA platform. Therefore, we can conclude that the variations observed between the drug responses of different patients (Figure 3) reflect true biological differences in drug sensitivity rather than technical inconsistencies in the assay.

#### 3.2.4 Enabling Drug Sensitivity Testing on Needle Biopsy Samples

So far, the application of drug sensitivity tests is largely limited to tumors obtained through surgical resection. This means that the current approach may primarily benefit lung cancer patients who undergo surgery. However, for patients with advanced‐stage cancers or metastasis, tissue samples are typically collected through needle biopsies, which yield a much smaller amount of cellular material [19]. This limited cell count poses a significant challenge, as it may not be sufficient for traditional drug sensitivity testing [8].

**FIGURE 5:**
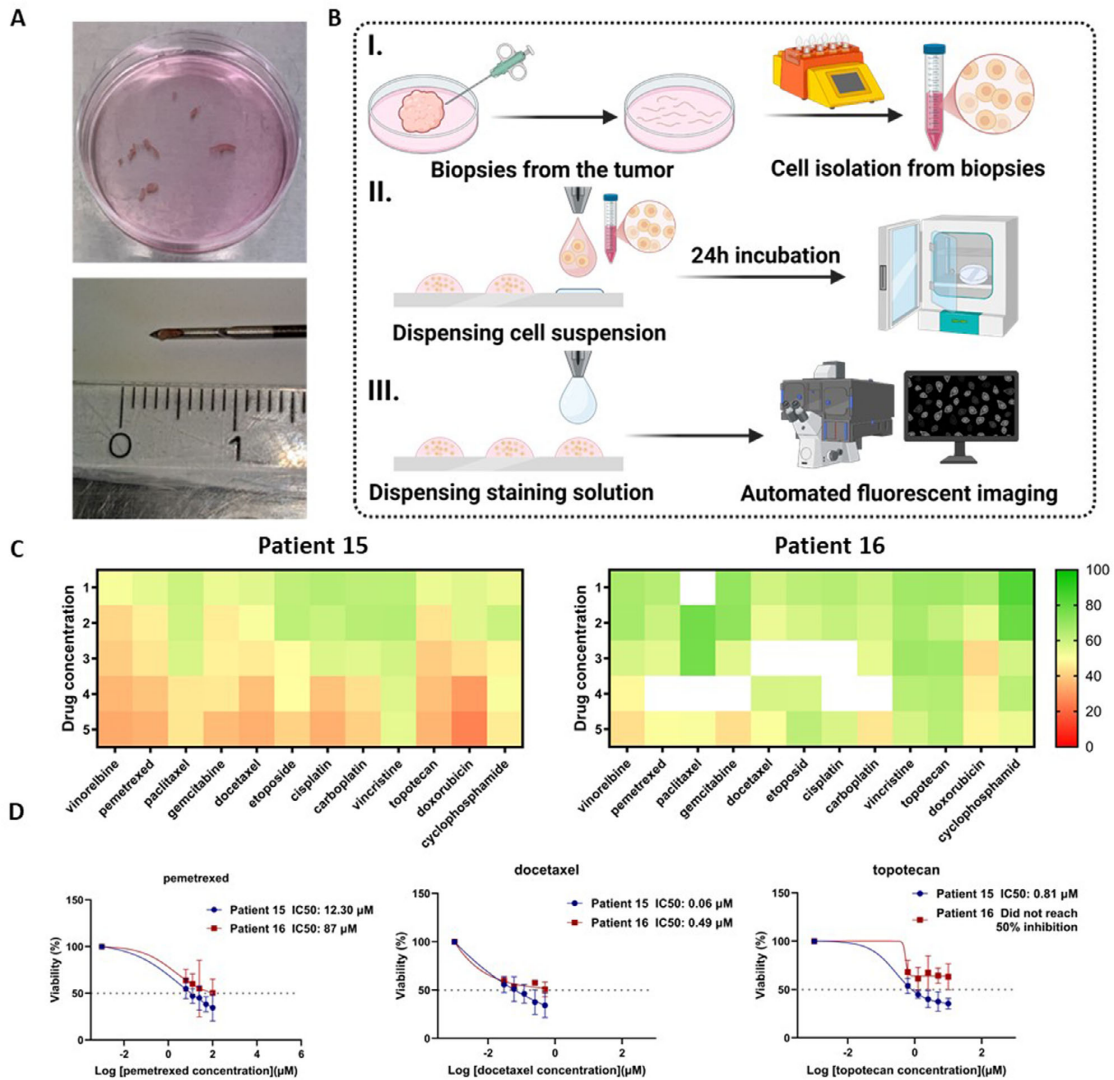
Drug sensitivity testing using small needle biopsy samples. (A) Examples of generated biopsies from a tumor (upper panel) and a close‐up showing an exemplary biopsy size (lower panel). (B) Schematic workflow of the drug sensitivity testing on needle biopsy samples. (C) Drug sensitivity results for the two artificial needle biopsy samples after 24 h of drug incubation. Cells were stained with a solution containing Hoechst 33342, Calcein‐AM, and PI. Due to a technical issue during image acquisition, some images for Patient 16 were lost, and image analysis was not possible for some drug concentrations, and remain empty. (D) Dose‐response curves generated using data from cell viability assays after 24 h. Viability percentages at different drug concentrations were plotted, and the curves were fitted using nonlinear regression analysis. The IC50 was calculated from five repeats, and error bars represent standard deviations.

To assess the feasibility of the DMA platform for conducting drug sensitivity testing on samples from late‐stage lung cancer, we generated artificial needle biopsies with a 21G needle used during clinical routine CT‐guided biopsy collection. A total of 8 artificial needle biopsies were obtained from each of three tumor specimens obtained from surgery (Patients 14, 15, and 16), with the combined weight of all 8 biopsies per patient measuring approximately 0.02 g (Figure 5A). We obtained approximately 170,000, 190,000, and 200,000 cells from patients 14, 15, and 16, respectively. Cells from patient 14 were isolated using a manual dissociation method, while cells from patients 15 and 16 were processed using a semi‐automated approach. Following manual dissociation, cells from patient 14 exhibited low viability (26%; Figure S2A) and were therefore excluded from further analyses. This reduced viability is consistent with the lower efficiency of the manual dissociation method, which, in our study, yielded fewer viable cells compared to the semi‐automated method. In contrast, cells from patients 15 and 16 showed viabilities of 84% and 81%, respectively, and were used for subsequent drug screening.

Figure 5D and Figure S10 show that we were able to obtain robust dose‐response curves for each drug. Figure 5D shows a comparison of the responses of biopsy‐derived cells from patients 15 and 16. Notably, the curves illustrate a differential response to topotecan, with patient 15 showing an IC50 of 0.81 µM, whereas patient 16 did not reach 50% inhibition. In this study, we did not compare drug responses between biopsy‐derived samples and matched surgically resected tumors, as this was beyond the scope of the current work and will be addressed in future studies aimed at clinical validation of the platform.

The total number of cells required to screen 12 compounds can be calculated as follows: for 300 cells per droplet, 5 concentrations plus a vehicle control, 5 replicates, and 12 drugs, the total is 300 × 6 × 5 × 12 = 108,000 cells. To achieve 300 cells in 200 nL droplets, the cell suspension needs a density of 1.5 × 10^6^ cells/mL. With 200,000 cells in total, this corresponds to a total volume of 133 µL. Using our low‐volume dispenser, which has a dead volume of approximately 5–10 µL, around 120 µL of this suspension can be effectively utilized for dispensing. The experiment testing 12 drugs required about 75 µL in total, well within the available volume. Therefore, the total of 190,000 cells obtained was sufficient for this study, and a total input of approximately 120,000 cells would likely have been adequate for performing the same experiment.

It is important to note that working with such minimal volumes is not feasible using conventional microtiter plate‐based systems. In this study, we have demonstrated that a panel of 12 drugs, with a relatively rigorous experimental design, including five concentrations, a vehicle control, and five replicates, can be tested using as few as 1000 cells, while still yielding robust and quantifiable drug response data. These results highlight the potential of the DMA platform to perform functional drug sensitivity testing on extremely limited clinical samples, such as needle biopsy‐derived material, at a scale that is inaccessible to standard screening approaches.

## 4 Discussion

This study presents a miniaturized drug sensitivity and resistance testing (DSRT) workflow using the DMA chip, addressing a key challenge in functional precision oncology. In many cases, patients receive anticancer treatments without prior knowledge of drug efficacy. This is especially critical in advanced‐stage lung cancer, where treatment decisions are time‐sensitive and based on limited biopsy material.

Conventional drug screening platforms, such as 96‐well plates, typically require around 10,000 cells per condition [49, 50], making them impractical for the limited number of primary cells typically obtained from core needle biopsies. Even in a miniaturized microplate format, 384‐well plate‐based assays usually require on the order of 1000 cells per condition. In contrast, our DMA platform requires only 300 cells per 200‐nL droplet, reducing cell input by over 30‐fold compared to 96‐well formats and an approximately three‐fold reduction compared to 384‐well plates, while reducing reagent usage by 500 to 150 times, respectively. Using only 200,000 cells, we screened 12 anticancer drugs across 5 concentrations and 5 replicates each, an assay scale unfeasible with standard formats. These features make the platform suitable for functional drug testing in real‐world clinical applications where only a small number of patient‐derived cells are available. This miniaturized approach significantly reduces costs by conserving compound libraries, enabling simultaneous testing of multiple drugs and concentration gradients, and allowing for a more comprehensive drug sensitivity profile in a single experiment. Furthermore, reproducibility assessments across different cell numbers per spot showed consistent viability and drug response across droplets containing 300 to 700 cells, supporting the robustness of the assay with reduced cellular input.

The concept of pre‐printed libraries on DMA enables off‐the‐shelf compound panels on chip, which are particularly useful for rapid functional testing. Once cells become available, they can be dispensed onto such pre‐printed libraries within a minute using a very small volume of cell suspension (as low as 100 ‐ 200 µL). In this study, we demonstrated the comparability of drug response metrics obtained using the DMA platform and a standard 384‐well plate protocol. Nevertheless, differences in drug responses between conventional plates and the DMA system may arise from several factors. First, cell density differs between formats, with the DMA typically employing approximately tenfold higher nominal cell densities compared with plates. Previous studies have shown that cell density alone can influence drug sensitivity for certain compounds [51]. Second, differences in proliferation rates resulting from distinct culture volumes may affect responses to drugs with specific mechanisms of action. Third, even at identical nominal concentrations, the absolute amount of drug per condition differs due to the large disparity in assay volumes. Fourth, the higher surface‐to‐volume ratio in nanoliter droplets may influence drug adsorption, evaporation dynamics, and local concentration gradients. Fifth, variations in diffusion, oxygenation, and the accumulation of secreted factors may further contribute to altered cellular responses. Importantly, these differences do not imply that one format is inherently more accurate than the other, but rather reflect distinct experimental regimes. A systematic investigation of these parameters, including assay volume, cell density, and absolute drug dose, will be required to fully understand their impact on drug responses and is part of our ongoing work.

Two tumor dissociation approaches were evaluated in this study to reflect the protocol optimization process during workflow development. The manual dissociation method was initially implemented due to its accessibility and low technical requirements, and subsequently compared with a semi‐automated approach to improve cell yield and standardization. These methods were compared at the level of cell viability to identify the most suitable workflow for downstream DSRT. A direct end‐to‐end comparison of drug response profiles generated from the same tumor specimen processed by both dissociation methods was beyond the scope of this initial feasibility study but represents an important next step for future validation, particularly to assess potential effects of dissociation‐induced differences in cellular composition. EpCAM staining revealed a higher percentage of epithelial cells in the dissociated suspensions compared to the original tumor tissue. This likely reflects selective dissociation or survival of epithelial cells during enzymatic and mechanical processing, whereas stromal and immune components may be underrepresented. Epithelial cells, including both tumor and non‐tumor populations, are more readily liberated during dissociation, while stromal elements often remain embedded within the extracellular matrix [52]. In this study, EpCAM staining was used to confirm the presence of epithelial cells after dissociation and incubation, rather than to distinguish tumor cells from normal epithelial cells. Pathological assessment of tumor cell content provides an indirect estimate of the expected proportion of malignant cells in the isolated cell population. The present study therefore, focused on establishing a feasible, low‐input DSRT workflow rather than investigating mechanistic contributions of individual tumor microenvironment components, which will be an important direction for future studies.

Conventional DSRT approaches often require in vitro expansion of patient‐derived cells to generate sufficient material for screening, a process that can take weeks to months of cell expansion, a time frame that many late‐stage patients cannot afford. By contrast, the DMA‐based workflow enables direct drug sensitivity testing on freshly isolated tumor cells using minimal input material, thereby bypassing the need for expansion. As a result, drug response data can be obtained within less than 1 week from tissue acquisition, a timeframe that is important for facilitating early treatment decisions in clinics for advanced‐stage lung cancer. This study also demonstrated that the DMA platform successfully captures interpatient variability in drug responses. For example, cells from different patients with the same lung cancer subtype exhibited varying sensitivity to doxorubicin. Additionally, the analysis of three distinct tumor regions revealed consistent drug responses within a particular sample. As none of the patients included in this study received chemotherapy post‐surgery, clinical outcome correlation was not possible. As a result, we could not assess the predictive value of the in vitro drug sensitivity profiles.

In this study, a scaffold‐free liquid‐media format was used as an initial approach for functional testing of freshly isolated primary lung cancer cells on the DMA platform. The patient‐derived cells grew as single cells or formed multicellular aggregates and did not adhere to the surface. Initially, we evaluated cell culture using the hanging‐drop method and hydrogel‐based scaffolds; however, no changes in cell morphology were observed compared to liquid culture. Therefore, for subsequent experiments, we continued using a liquid medium, which also offered greater experimental simplicity.

The native 3D tissue architecture is intentionally disrupted during tissue dissociation to release individual cells for functional testing. While this scaffold‐free approach does not recapitulate the full tumor architecture or in vivo microenvironmental dynamics, it preserves the cellular composition of the tumor by enabling testing of the complete mixture of isolated cells. Importantly, this liquid‐based approach allows rapid and reproducible drug sensitivity testing of non‐expanded primary cells under clinically relevant constraints of limited material and time.

By addressing practical limitations of conventional drug sensitivity and resistance testing (DSRT) methods, most notably high cell and reagent requirements, this miniaturized approach provides a feasible framework for functional drug testing using limited biopsy‐derived material. The DMA‐based workflow enables drug sensitivity assessment on freshly isolated patient‐derived cells without prior in vitro expansion and supports reproducible screening under low‐input conditions.

While this study represents an initial demonstration of DSRT for solid tumors on the droplet microarray platform rather than the introduction of a new assay principle, it establishes a materials‐enabled workflow that allows quantitative functional testing in a regime that is difficult to access with conventional formats. In particular, the ability to generate reproducible drug response profiles directly from small solid tumor samples highlights the potential of droplet‐based microarrays to extend functional precision oncology approaches to biopsy‐limited clinical settings.

Future efforts will also focus on comprehensive characterization of the tumor‐derived cellular composition, including immune cell populations, to better reflect tumor complexity and improve the predictive value of the assay. Such characterization may enable identification of drug‐sensitive cell subpopulations and support the potential in vitro evaluation of immune checkpoint inhibitors, which requires the presence of functional immune components.

The envisioned clinical implementation of the DMA platform relies on off‐the‐shelf, ready‐to‐use slides pre‐printed with a fixed panel of clinically relevant drugs. Unlike discovery‐scale screening, ultra–high‐throughput testing of thousands of compounds is neither necessary nor practical in a diagnostic setting. A single DMA chip accommodates approximately 550 experimental points, allowing the testing of ∼50 drugs per patient, which is well aligned with clinical decision‐making and suggests that one chip per patient would typically be sufficient. Within such a workflow, clinical adoption would require only cell dispensing followed by incubation. While a certain degree of automation will be necessary, particularly for evaporation control, this can be readily achieved through automated humidity regulation during transfer between the dispenser, incubator, and imaging systems. The development of such automated handling and environmental control systems is technically straightforward once platform performance is established and would primarily depend on funding rather than technological limitations.

Further validation with larger cohorts and systematic comparison with clinical outcomes will be required to assess predictive value. Nevertheless, the present results define a methodological foundation for integrating miniaturized functional assays into future translational and clinical workflows, where tissue availability and turnaround time are critical constraints.

## Conflicts of Interest

The authors declare no conflicts of interest.

## Supporting information




**Supporting File**: adhm71256‐sup‐0001‐SuppMat.docx.

## Data Availability

The data that support the findings of this study are available at https://doi.org/10.35097/nnceq5ew75kw8124.
