# Supplementary material for: Functional Precision Oncology Approach Using Nanoliter Droplet Array for Drug Sensitivity Testing in Lung Cancer
Source: Adv Healthc Mater. 2026 May 15;15(24):e03761. doi: 10.1002/adhm.202503761 (PMC13307630; doi:10.1002/adhm.202503761)
Supplement: Supplementary file 1 — Supporting File: adhm71256‐sup‐0001‐SuppMat.docx. [file ADHM-15-0-s001.docx]

**Supplementary**

| **Patient ID** | **Number of isolated cells (x10^6^)** | **Cell viability after isolation (%)** | **Cell isolation method** | **Tumor volume (cm3)** | **Normalized number of isolated cells** |
| --- | --- | --- | --- | --- | --- |
| Patient 1 | 13.8 | 90 | Semi-automated | 3.6 | 3.83 |
| Patient 2-1 | 1.26 | 67 | Semi-automated | 0.23 | 5.48 |
| Patient 2-2 | 1.28 | 73 | Semi-automated | 0.3 | 4.27 |
| Patient 2-3 | 0.91 | 84 | Semi-automated | 0.18 | 5.06 |
| Patient 3 | 6.35 | 86 | Semi-automated | 1.87 | 3.40 |
| Patient 4 | 6.9 | 98 | Semi-automated | 1.1 | 6.27 |
| Patient 5 | 2.3 | 78 | Semi-automated | 1.01 | 2.28 |
| Patient 6 | 1.47 | 58 | Manual | 1 | 1.47 |
| Patient 7 | 1.49 | 89 | Manual | 1.17 | 1.27 |
| Patient 8 | 1.83 | 65 | Manual | 1.35 | 1.36 |
| Patient 9 | 2.1 | 83 | Manual | 0.9 | 2.33 |
| Patient 10 | 1.64 | 69 | Manual | 0.9 | 1.82 |
| Patient 11 | 4.2 | 72 | Manual | 1.2 | 3.50 |
| Patient 12-1 | 0.71 | 61 | Semi-automated | 0.17 | 4.18 |
| Patient 12-2 | 0.93 | 59 | Semi-automated | 0.23 | 4.04 |
| Patient 12-3 | 1.21 | 53 | Semi-automated | 0.31 | 3.90 |
| Patient 13-1 | 0.92 | 74 | Semi-automated | 0.32 | 2.88 |
| Patient 13-2 | 2.7 | 88 | Semi-automated | 0.4 | 6.75 |
| Patient 13-3 | 3.87 | 64 | Semi-automated | 0.41 | 9.44 |

**Supplementary Table 1. Tumor dissociation results for each patient.** The table includes total number of viable cells isolated, viability of isolated cells (%), the method of cell isolation, estimated tumor volume (cm³), and normalized cell yield (viable cells per cm³ of tumor tissue) for each sample. Tumor volume was calculated as length × width × height.

**
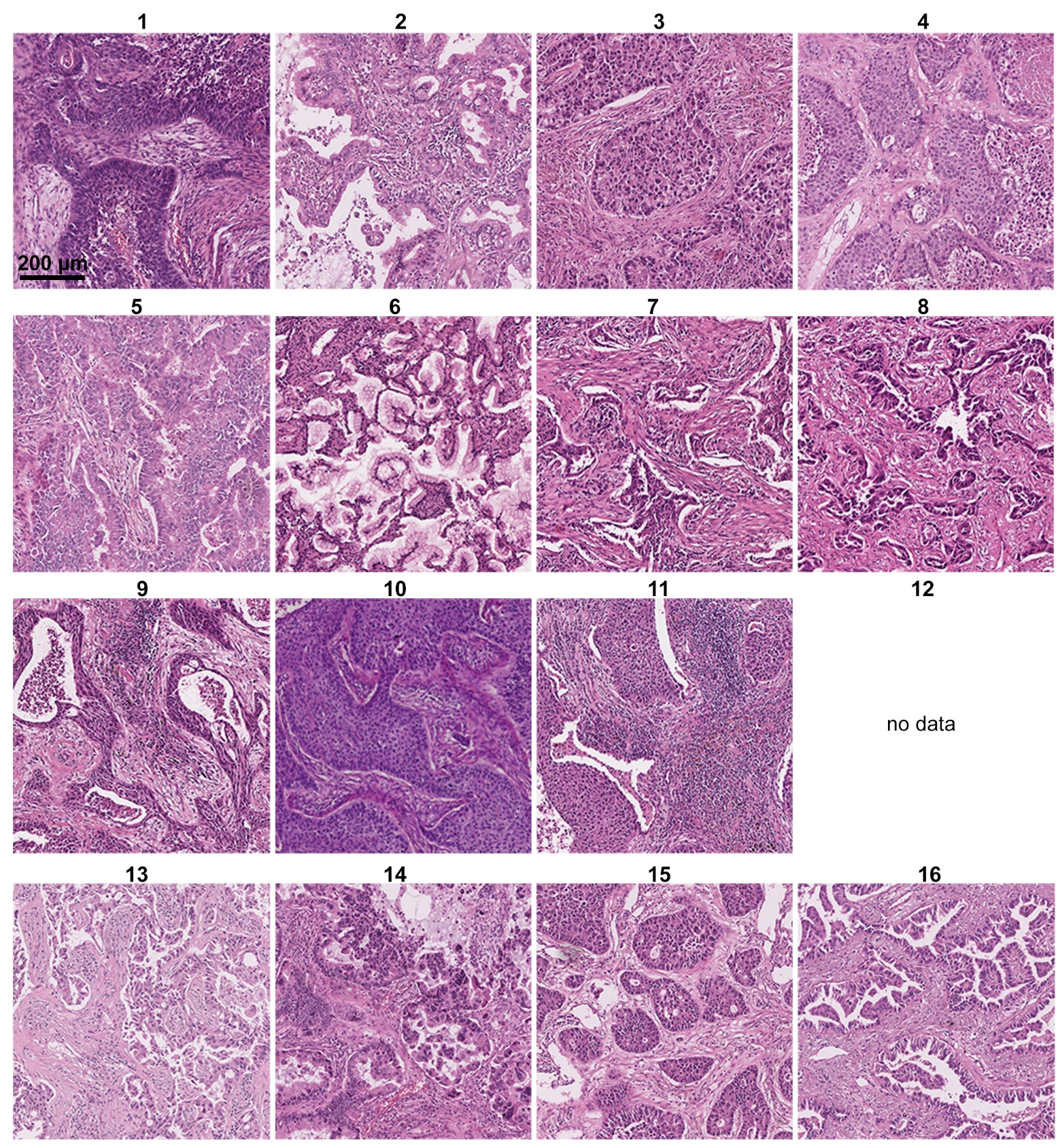
**

**Supplementary Figure 1. H&E staining of samples used in this study.**


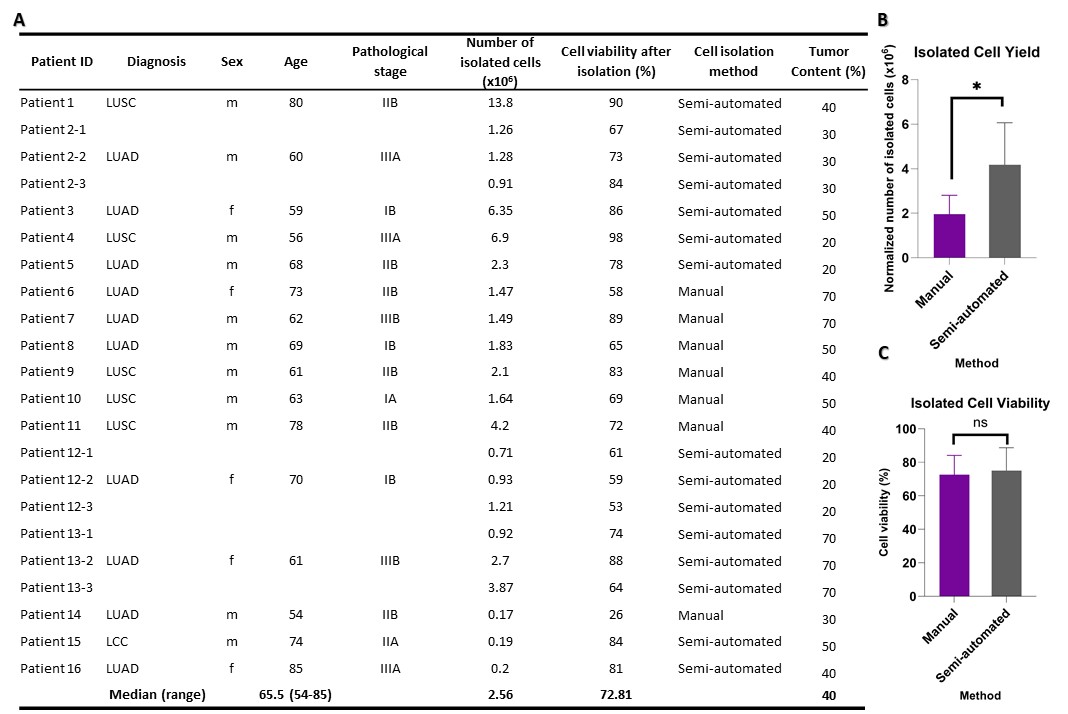


**Supplementary Figure 2.** **Overview of patient-derived lung tumor samples and cell isolation outcomes.** A) List of patient-derived tumor samples used in the study. For each patient, tumor type, number of viable isolated cells (in millions), post-isolation cell viability (%) and tumor content (%) are provided. Tumor subtypes are indicated as LUAD (lung adenocarcinoma), LUSC (lung squamous cell carcinoma), and LCC (large cell carcinoma). Patient sex is indicated as m (male) or f (female). Patients are listed according to their appearance in the results section. Samples from Patients 2, 12, and 13 were each divided into three sections, and cells were isolated from each section separately. The number and viability of isolated cells for each section are provided. Eight artificial needle biopsies were obtained from the samples of patients 14, 15, and 16, which were dissociated into cell suspensions. The number of isolated cells and their viability for each sample are reported. B) Comparison of the normalized number of isolated cells between manual and semi-automated tumor dissociation methods. Error bars represent the standard deviation. A statistically significant difference was observed (unpaired t-test, p = 0.014). C) Comparison of post-isolation cell viability between manual and semi-automated tumor dissociation methods. Error bars represent the standard deviation. No statistically significant difference was observed (unpaired t-test, p = 0.72).


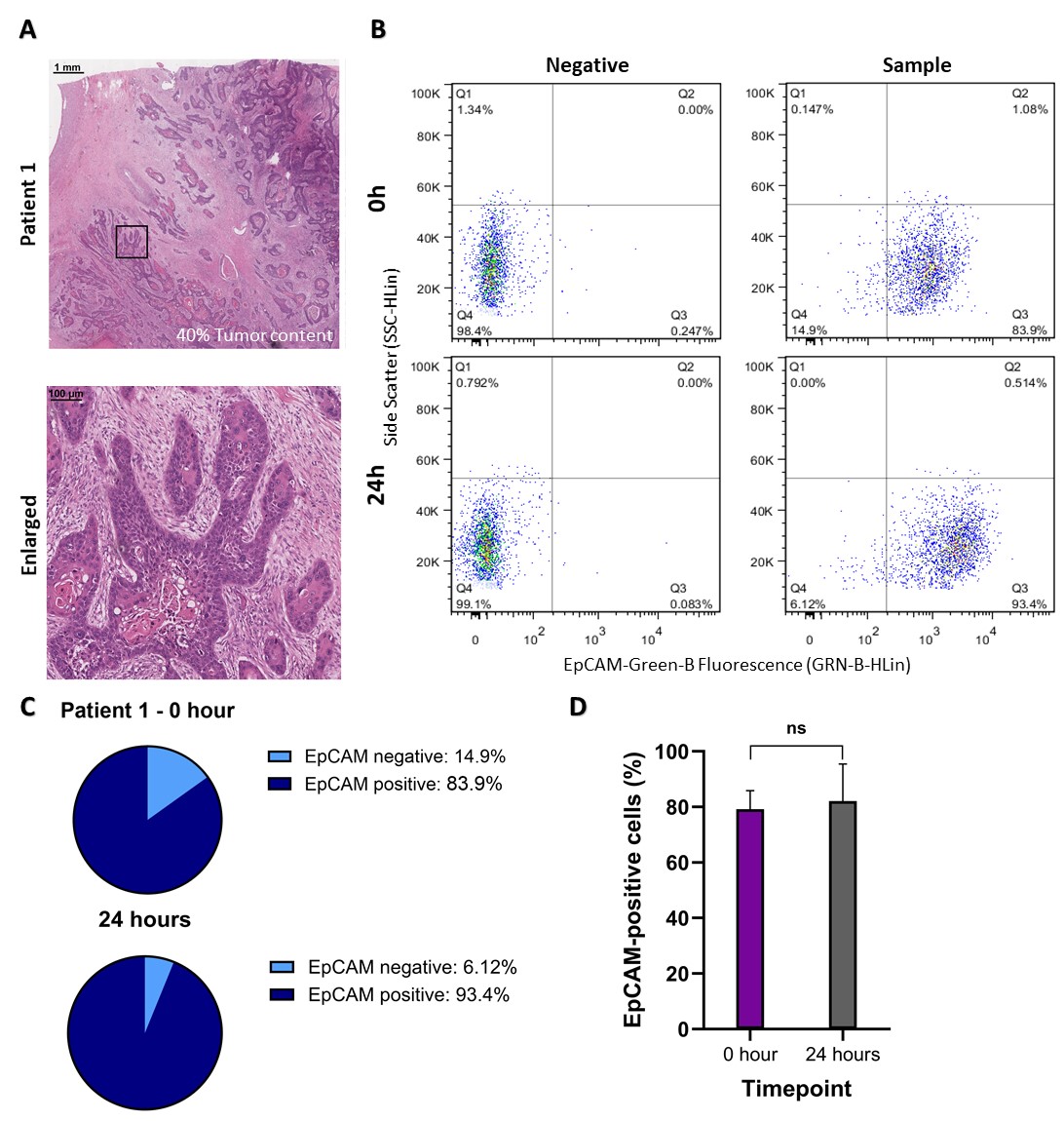


**Supplementary Figure 3.** **Detection of EpCAM-positive cells in cell suspensions post-isolation and after 24-hour incubation.** A) Exemplary H&E staining of the tumor sample from Patient 1. B) flow cytometry analysis of EPCAM-positive and -negative cells from patient 1. Cells were stained with an Alexa Fluor® 488-conjugated anti-EpCAM antibody. Cell populations were graphed as side scatter vs. Green B fluorescence, with Q4 representing the EpCAM-negative and Q3 the EpCAM-positive population. Negative controls without anti-EpCAM antibody staining were included for each time point. C) The pie chart summarizes the results obtained from Patient 1 after immediate dissociation and 24 hours of incubation. D) The percentage of EpCAM-positive cells was measured immediately after tumor dissociation and after 24 hours of culture. Bars represent the mean ± standard deviation (SD) across three patient-derived samples. Statistical comparison was performed using an unpaired t-test; no significant difference was observed (p = 0.7529).

**
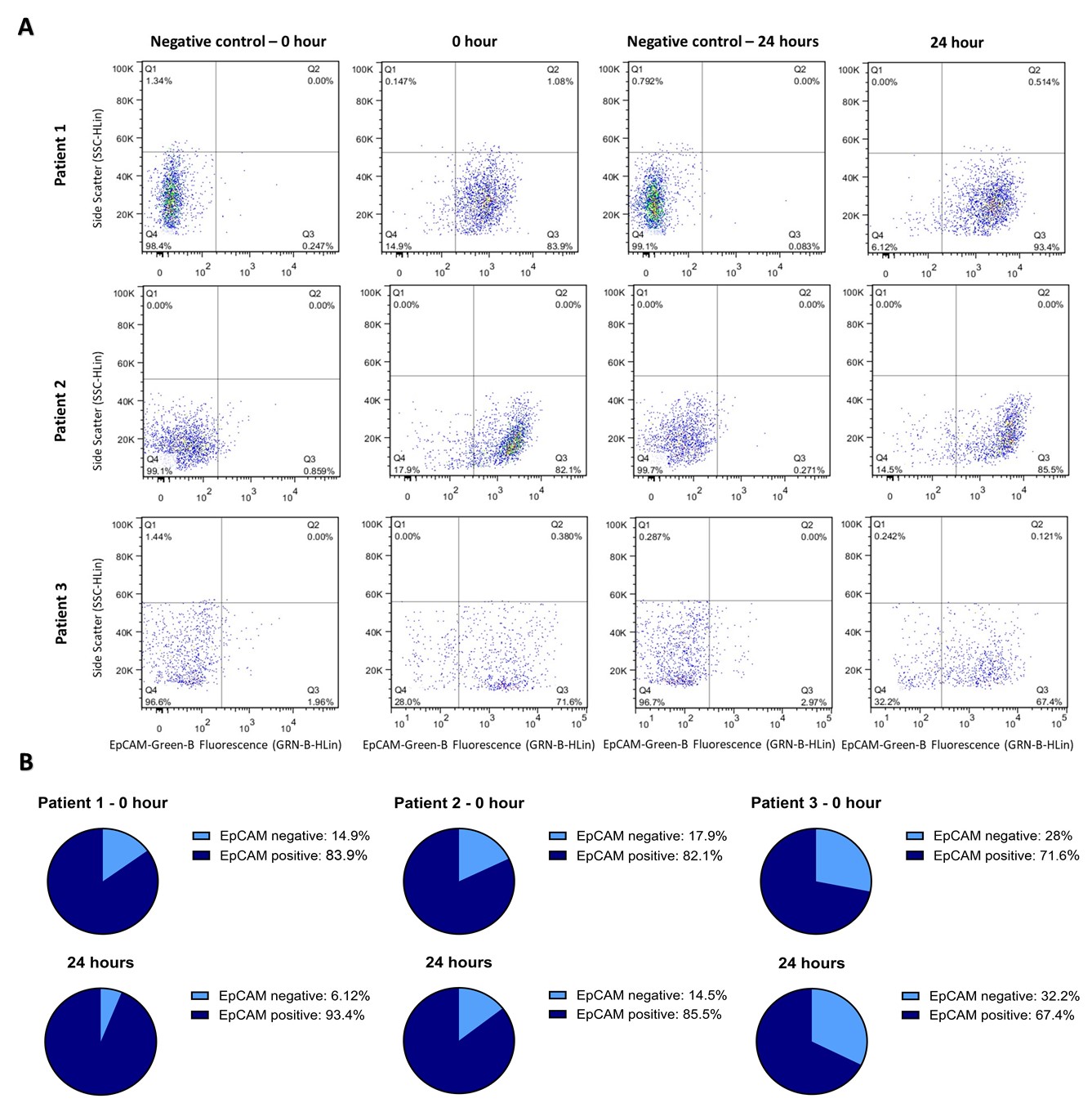
**

**Supplementary Figure 4. Detection of EpCAM-positive cells in cell suspensions post-isolation and after 24-hour incubation.** A) Cells obtained from samples (Patient 1, 2 and 3) were stained with a conjugated anti-EpCAM antibody detected in the Green B fluorescent channel. Cell populations were graphed as side scatter vs. Green B fluorescence, where Q4 represents the EpCAM-negative population and Q3 represents the EpCAM-positive population. Negative controls, without anti-EpCAM antibody staining, were included for each time point. B) The pie charts summarize the results obtained from Patient 1, 2 and 3 after immediate dissociation (Upper charts) and after 24 hours of incubation (lower charts).

**
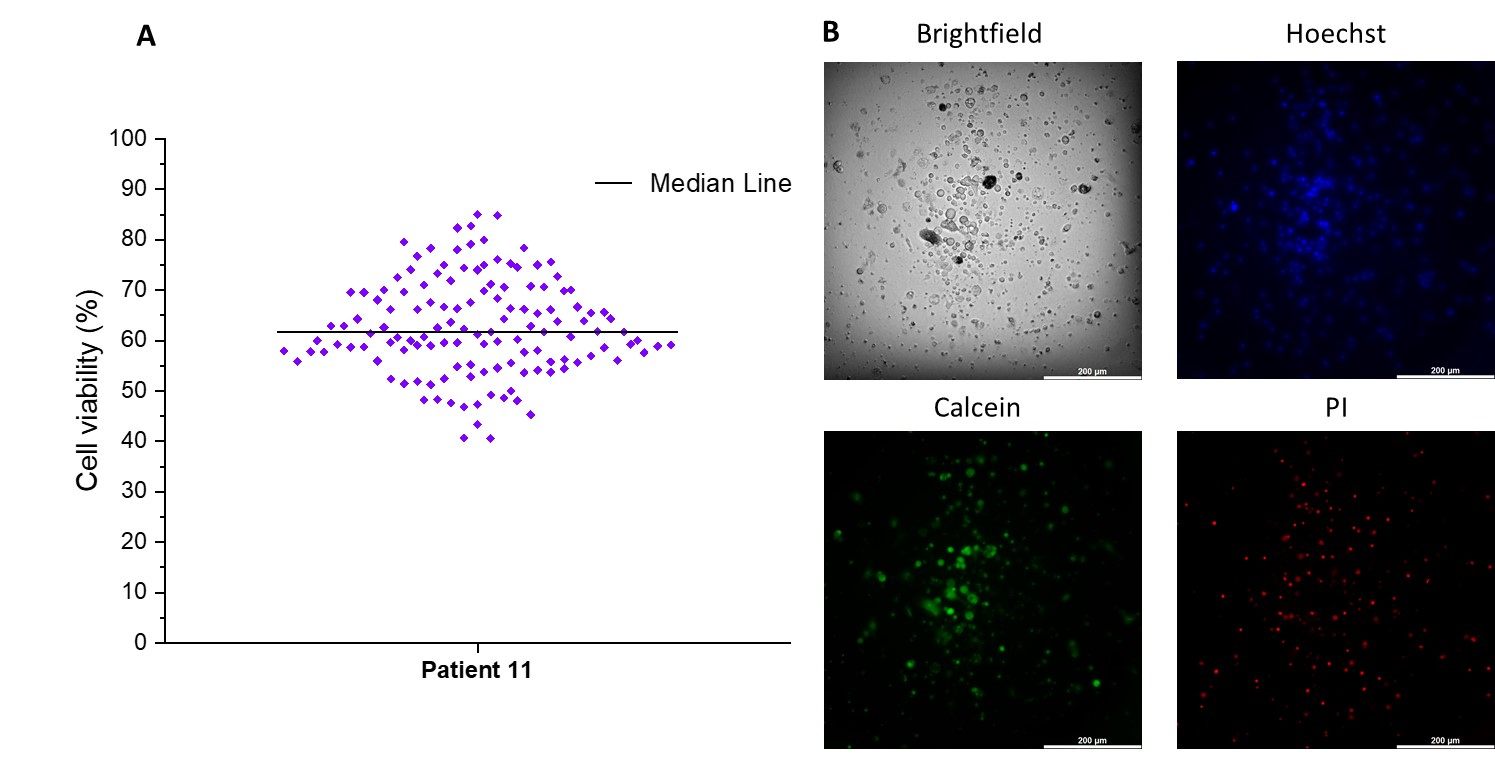
**

**Supplementary Figure 5. Cell viability of isolated tumor cells cultured on the DMA chip.** A) A total of 300 cells per 200-nanoliter droplet were cultured on 134 spots of the DMA chip. After 24 hours of incubation, cells were stained with Hoechst 33342, Calcein-AM, and PI. Cell viability was calculated as the ratio of Calcein-positive cells to the total number of cells (Calcein-positive + PI-positive). B) Representative images of a single DMA spot containing isolated tumor cells stained with Hoechst 33342, Calcein-AM, and PI. Scale bar: 200 μm.

**
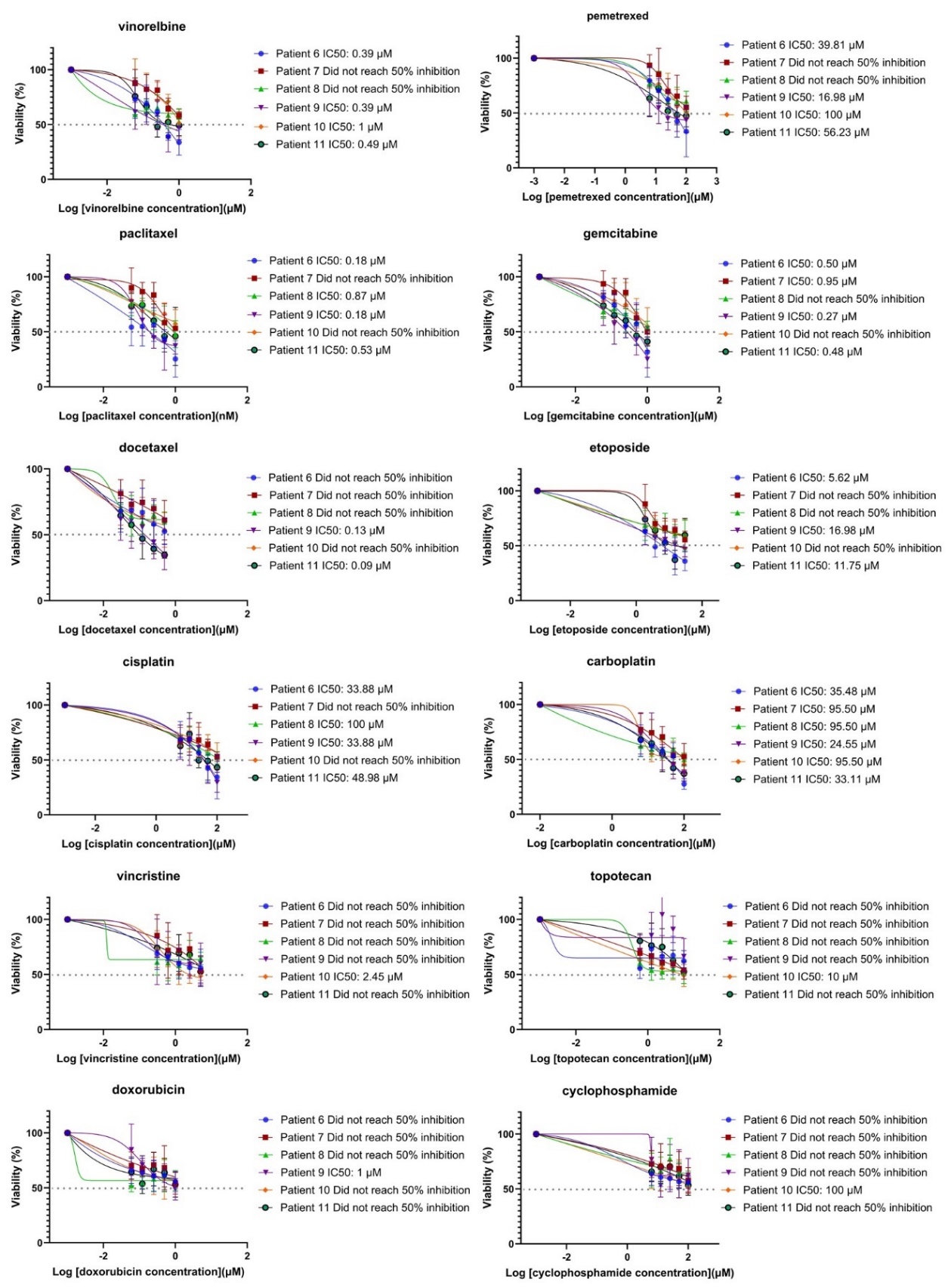
**

**Supplementary Figure 6. Comparison of dose-dependent effects of compounds on primary patient-derived cells cultured on a DMA chip.** A total of 300 cells were cultured in 200-nanoliter droplets and exposed to drugs for 24 hours. After incubation, cells were stained with Hoechst 33342, Calcein-AM, and PI, and imaged using an automated fluorescence microscope. The IC50 of each drug was determined. The average was taken from five repeats, with error bars indicating standard deviations.


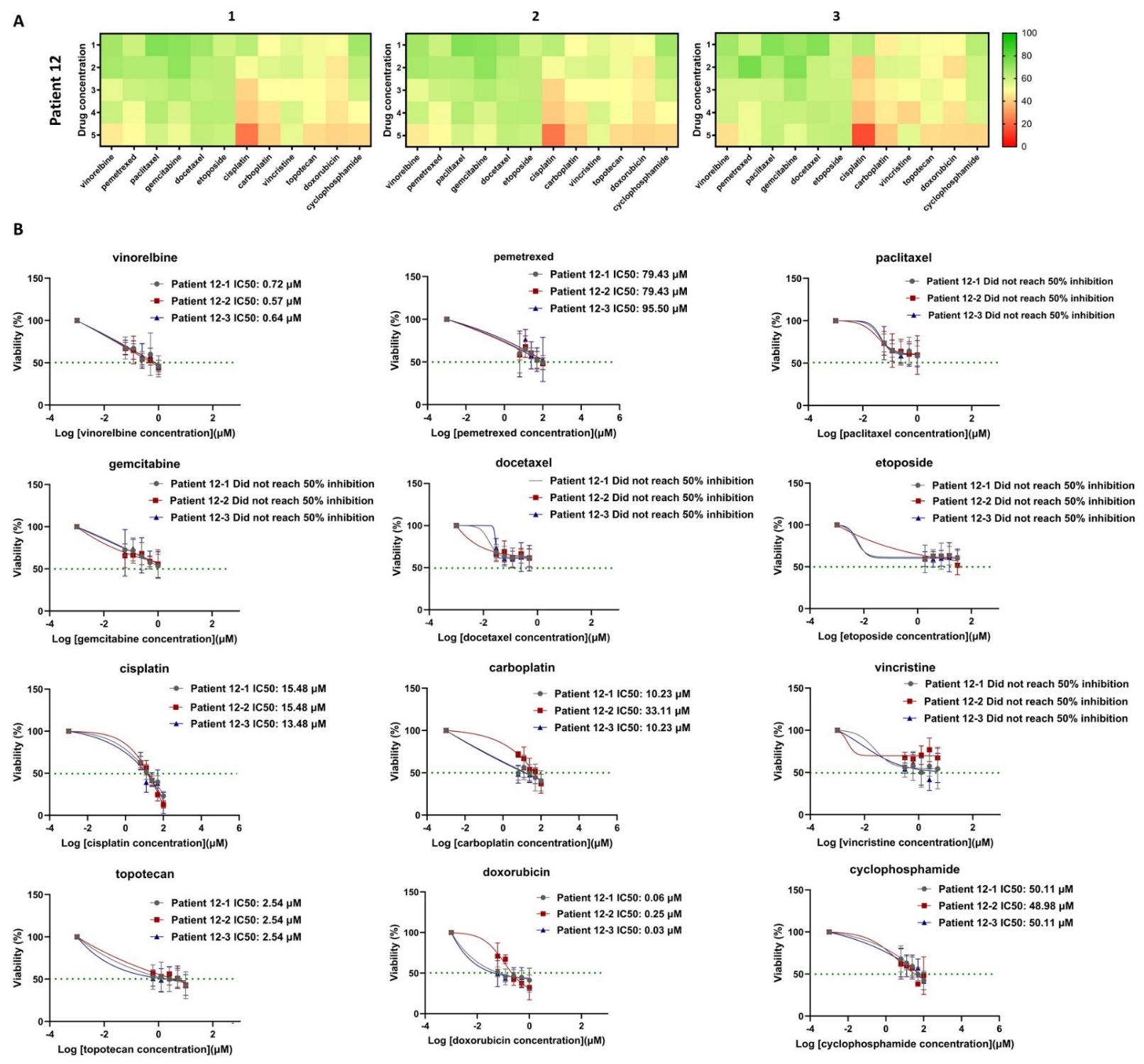


**Supplementary Figure 7.** **Dose–Response Curves from Distinct Tumor sections of Patient 12.** A) Each heatmap represents the drug responses of one tumor section: left (1), center (2), and right (3). Drug sensitivity was assessed after 24 hours of incubation. Cells were stained with a solution containing Hoechst 33342, Calcein-AM, and PI. Drug concentration 1 represents the lowest concentration, and Drug concentration 5 represents the highest. Green spots indicate cell viability greater than 60%, yellow spots indicate viability between 60% and 40%, and red spots indicate viability below 40%. B) Dose-response curves generated using data from cell viability assays after 24 hours of drug incubation from cells isolated from Patient 12's specimen. Viability percentages at different drug concentrations were plotted, and the curves were fitted using nonlinear regression analysis. The IC50 for each section was determined using the curves. The average was calculated from five repeats, and error bars represent standard deviations.


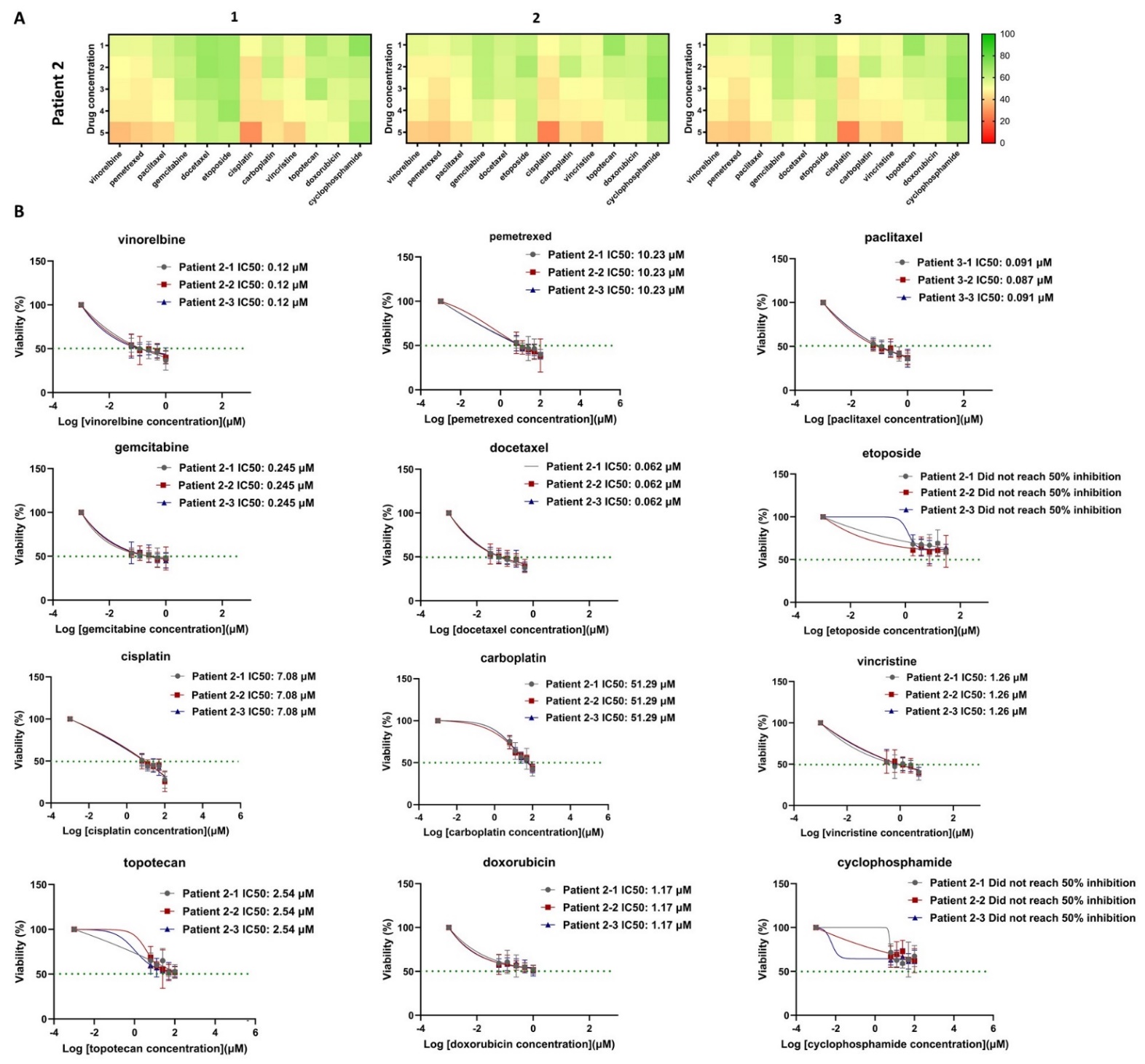


**Supplementary Figure 8.** **Dose–Response Curves from Distinct Tumor sections of Patient 2.** A) Each heatmap represents the drug responses of one tumor section: left (1), center (2), and right (3). Drug sensitivity was assessed after 24 hours of incubation. Cells were stained with a solution containing Hoechst 33342, Calcein-AM, and PI. Drug concentration 1 represents the lowest concentration, and Drug concentration 5 represents the highest. Green spots indicate cell viability greater than 60%, yellow spots indicate viability between 60% and 40%, and red spots indicate viability below 40%. B) Dose-response curves generated using data from cell viability assays after 24 hours of drug incubation from cells isolated from Patient 2's specimen. Viability percentages at different drug concentrations were plotted, and the curves were fitted using nonlinear regression analysis. The IC50 for each section was determined using the curves. The average was calculated from five repeats, and error bars represent standard deviations.

**
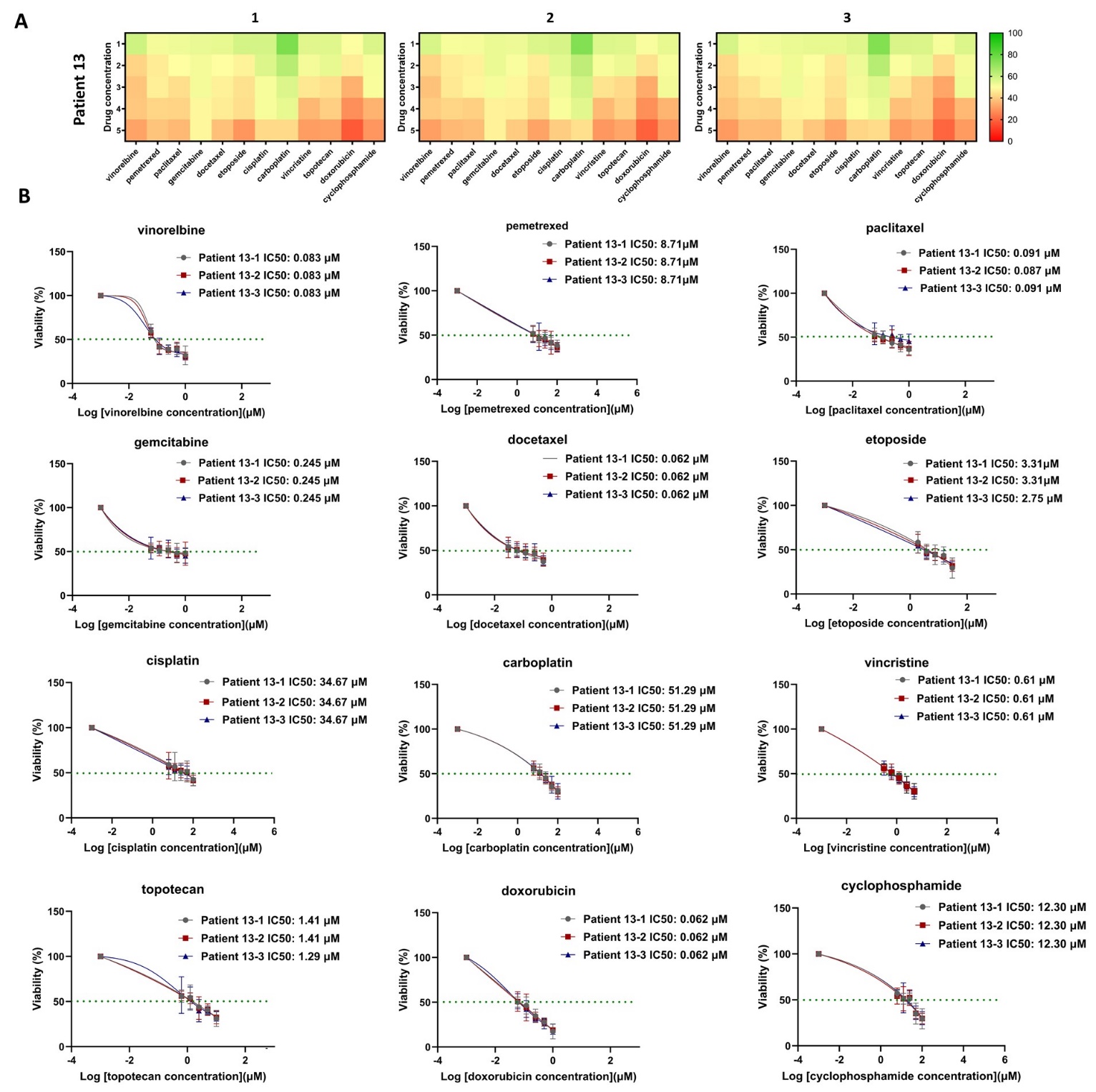
**

**Supplementary Figure 9.** **Dose–Response Curves from Distinct Tumor sections of Patient 13.** A) Each heatmap represents the drug responses of one tumor section: left (1), center (2), and right (3). Drug sensitivity was assessed after 24 hours of incubation. Cells were stained with a solution containing Hoechst 33342, Calcein-AM, and PI. Drug concentration 1 represents the lowest concentration, and Drug concentration 5 represents the highest. Green spots indicate cell viability greater than 60%, yellow spots indicate viability between 60% and 40%, and red spots indicate viability below 40%. B) Dose-response curves generated using data from cell viability assays after 24 hours of drug incubation from cells isolated from Patient 13's specimen. Viability percentages at different drug concentrations were plotted, and the curves were fitted using nonlinear regression analysis. The IC50 for each section was determined using the curves. The average was calculated from five repeats, and error bars represent standard deviations.

**
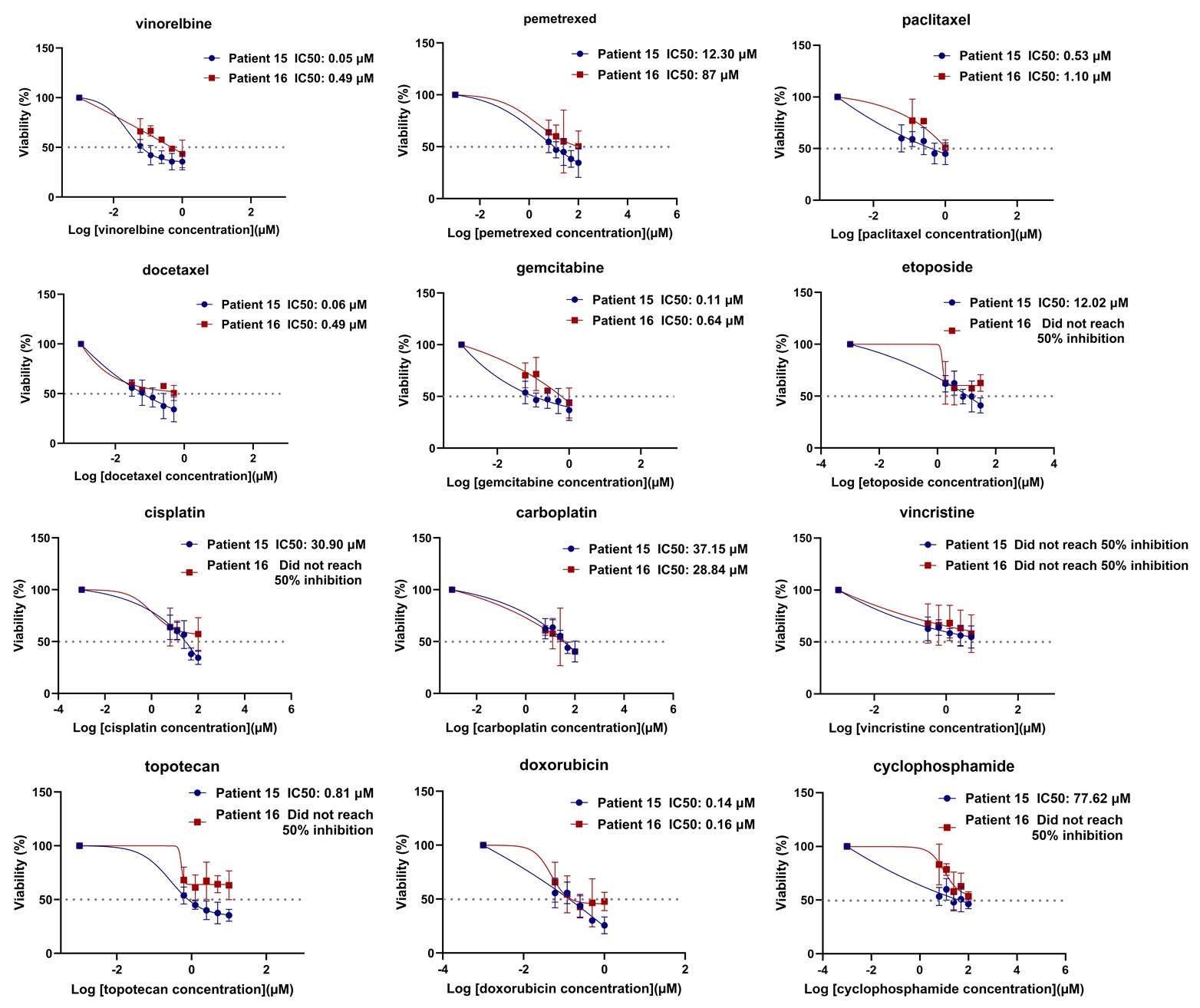
**

**Supplementary Figure 10.** **Dose-response curves generated using data from cell viability assays after 24 hours of drug incubation from cells isolated from Patient 15's and Patient 16's artificial needle biopsies.** Viability percentages at different drug concentrations were plotted, and the curves were fitted using nonlinear regression analysis. The IC50 of for both samples were determined using the curves. The average was calculated from five repeats, and error bars represent standard deviations.


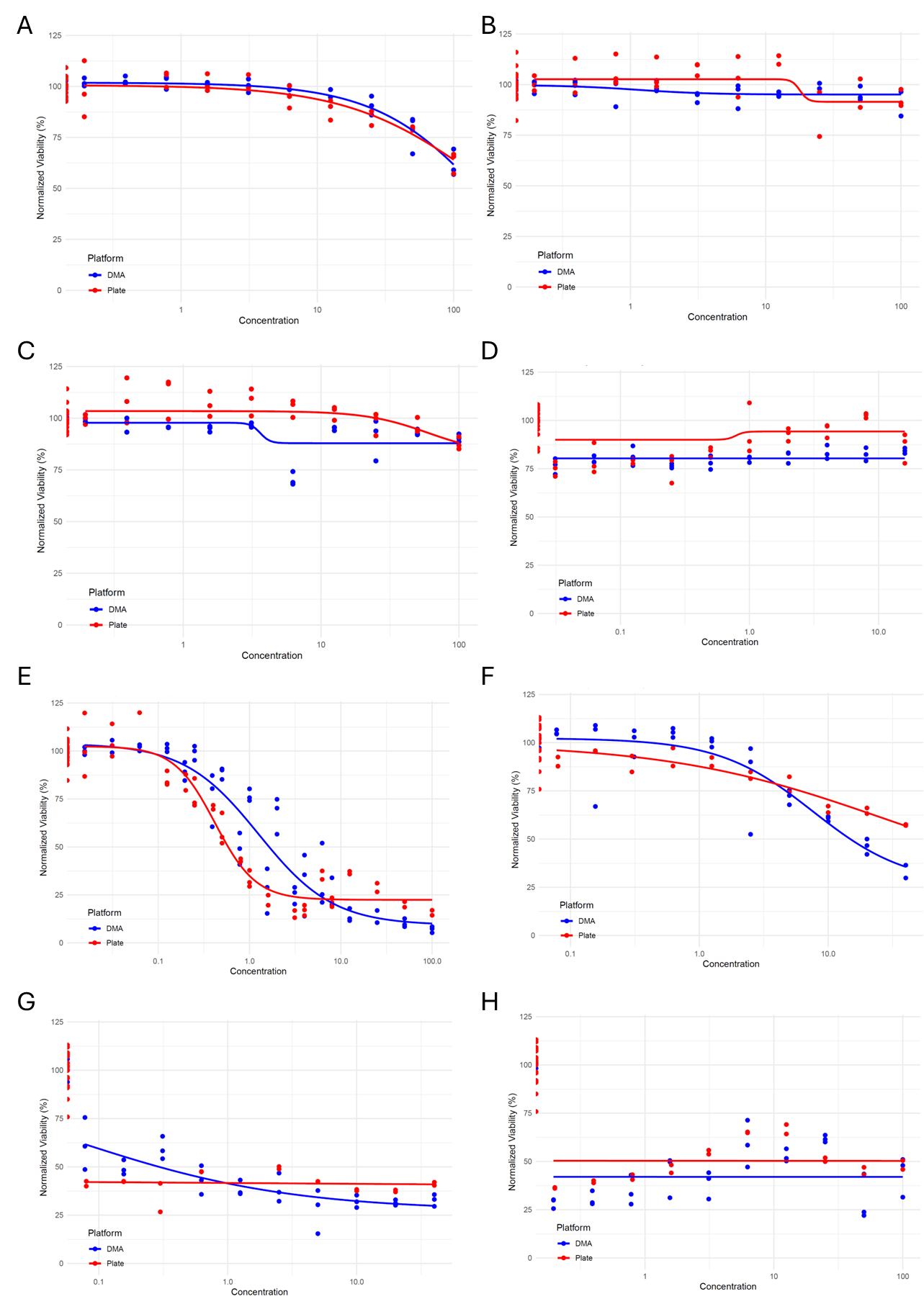

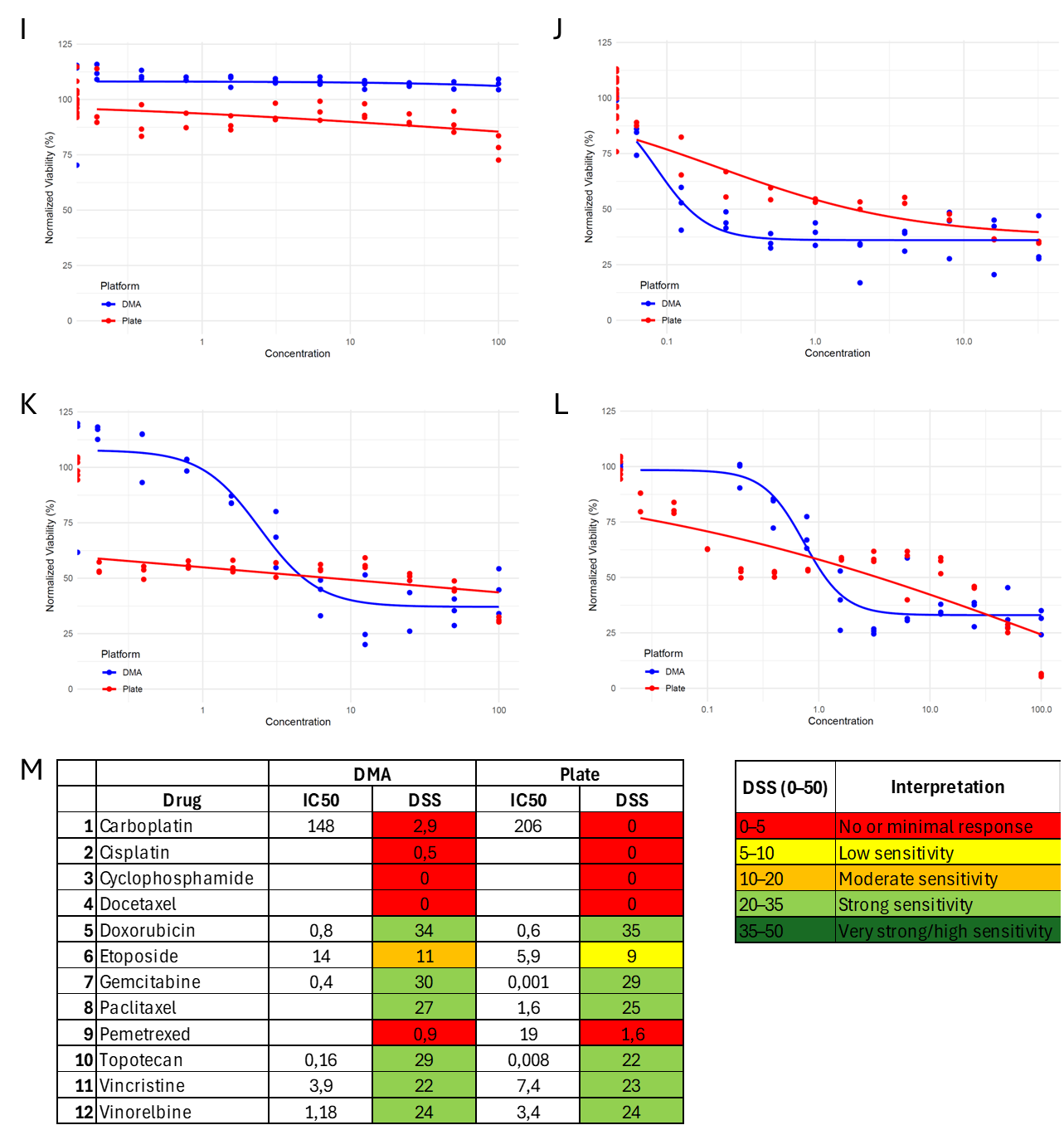


**Supplementary Figure 11.** **Comparison of dose–response profiles of 12 drugs evaluated using DMA and 384-well plate platforms**. The following compounds were tested: carboplatin (A), cisplatin (B), cyclophosphamide (C), docetaxel (D), doxorubicin (E), etoposide (F), gemcitabine (G), paclitaxel (H), pemetrexed (I), topotecan (J), vincristine (K), and vinorelbine (L). All drugs were assessed on DMA using pre-printed and dried compound libraries, as applied in this study, and in 384-well plates using the state-of-the-art CellTiter-Glo viability assay. Panels A–L show the comparison of dose–response curves obtained from the DMA and 384-well plate platforms. (M) Table summarizing the comparison of IC₅₀ and DSS (Drug Sensitivity Score) values for both platforms. The interpretation of DSS annotations is provided in the table on the right.
